# Hyperammonemia induces programmed liver cell death

**DOI:** 10.1126/sciadv.ado1648

**Published:** 2025-03-07

**Authors:** Annarein J. C. Kerbert, Cornelius Engelmann, Abeba Habtesion, Pavitra Kumar, Mohsin Hassan, Tingting Qi, Ines Volkert, Tobias Otto, Andrew Hall, Varun U. Khetan, Steven Olde Damink, Ferran Aguilar, Céline Chollet, Ludovic Brunet, Joan Clària, Richard Moreau, Vicente Arroyo, Minneke J. Coenraad, Gautam Mehta, Florence Castelli, Christian Trautwein, François Fenaille, Fausto Andreola, Rajiv Jalan

**Affiliations:** ^1^ Liver Failure Group, Institute for Liver and Digestive Health, University College London, Royal Free Campus, London, UK.; ^2^ Department of Gastroenterology & Hepatology, Leiden University Medical Center, Leiden, Netherlands.; ^3^ Medical Department, Division of Hepatology and Gastroenterology, Campus Virchow-Klinikum, Charite - Universitätsmedizin Berlin, Berlin, Germany.; ^4^ Department of CardioMetabolic Disease Research, Boehringer Ingelheim, Biberach, Germany.; ^5^ Department of Hepatology Unit and Infectious Diseases, Nanfang Hospital, Southern Medical University, Guangzhou, China.; ^6^ Department of Medicine III, University Hospital RWTH Aachen, Aachen, Germany.; ^7^ The Sheila Sherlock Liver Centre, Royal Free Hospital, London, UK.; ^8^ Department of Cellular Pathology, Royal Free Hospital, London, UK.; ^9^ Department of Surgery, NUTRIM School of Nutrition and Translational Research in Metabolism, Maastricht University, Maastricht, Netherlands.; ^10^ European Foundation for the Study of Chronic Liver Failure, Barcelona, Spain.; ^11^ Université Paris-Saclay, CEA, INRAE, Département Médicaments et Technologies pour la Santé (DMTS), MetaboHUB-IDF, 91191 Gif-sur-Yvette, France.; ^12^ Hospital Clínic-IDIBAPS, University of Barcelona, Barcelona, Spain.; ^13^ Inserm and Université de Paris, Centre de Recherche sur l’Inflammation (CRI), UMRS1149 Paris, France.; ^14^ Service d’Hépatologie, Hôpital Beaujon, Assistance Publique-Hôpitaux de Paris, Clichy, France.; ^15^ The Roger Williams Institute of Hepatology, Foundation for Liver Research, London, UK.; ^16^ IFADO, Department of Toxicology, TU Dortmund, Dortmund, Germany.

## Abstract

Hyperammonemia is common in liver cirrhosis and causally associated with hepatic encephalopathy development. Little is known about its hepatotoxic effects, which we aimed to characterize in this study. In a mouse model of chronic hyperammonemia without preexisting liver disease, we observed development of liver fibrogenesis and necroptotic cell death. Hyperammonemia also induced dysregulation of its main metabolic pathway, the urea cycle, as reflected by down-regulation of urea cycle enzyme protein expression and accumulation of its metabolites. Inhibition of receptor-interacting serine/threonine-protein kinase 1 (RIPK1) and its upstream inducer Toll-like receptor 4 (TLR4) protected against liver injury and further hyperammonemia. In clinically relevant rodent models of hyperammonemia (genetic ornithine transcarbamylase deficiency and bile duct ligation–induced cirrhosis), TLR4 inhibition reduced circulating ammonia. In conclusion, hyperammonemia induces liver fibrogenesis and RIPK1-mediated cell death, which is associated with urea cycle dysfunction. Inhibition of RIPK1 and TLR4 protects against hyperammonemia-induced liver injury and are potential therapeutic targets for hyperammonemia and chronic liver disease progression.

## Teaser

Hyperammonemia induces liver injury, which can be prevented by inhibiting the Toll-like receptor 4 and RIPK1 pathway.

## INTRODUCTION

Ammonia is a nitrogenous metabolite that is mainly derived from intestinal bacterial production and amino acid (AA) metabolism. Under healthy conditions, ammonia is metabolized in the liver by the urea cycle and the enzyme glutamine synthetase (GS) (*1*). In liver cirrhosis and urea cycle enzyme (UCE) disorders (UCDs), these metabolic pathways are significantly impaired, which leads to hyperammonemia (*2*–*5*). Hyperammonemia is central in the pathophysiology of hepatic encephalopathy (HE) (*6*), a severe complication of liver disease and UCDs, which can manifest with a wide spectrum of symptoms ranging from subclinical alterations to coma (*7*). There are several mechanisms through which ammonia exerts its direct neurotoxic effects, involving several cell types in the brain and resulting in glutamine-related astrocyte swelling, oxidative stress, neuroinflammation, disturbed neurotransmitter homeostasis, and mitochondrial dysfunction (*8*).

It is therefore not unexpected that, more recently, studies revealed that ammonia is more than a neurotoxin and that it induces immune dysfunction (*9*–*11*) and sarcopenia (*12*–*16*). In addition, ammonia has been shown to activate hepatic stellate cells (HSCs), thereby worsening fibrosis and portal hypertension (*17*). There are supporting observations of the potential hepatotoxic effects of ammonia in cohorts of patients without underlying liver disease but with hyperammonemia due to inherited UCDs. Histopathological studies have shown evidence of fibrosis and cirrhosis development in patients with UCDs at later ages (*18*). This ability of ammonia to induce multiorgan toxicity may explain why circulating ammonia levels hold important prognostic information for both clinically stable outpatients (*19*) and hospitalized patients with cirrhosis (*20*–*22*), regardless of the presence of HE. Together, these data suggest that ammonia itself may act as a hepatotoxin that could further drive liver disease progression in cirrhosis and UCDs. This may have important implications for ammonia as a therapeutic target for the prevention of liver disease progression. However, it is currently unknown whether ammonia independently induces liver injury and what the underlying mechanisms are.

The current study aims at characterizing the hepatotoxic effects of ammonia in rodent models of “pure” hyperammonemia (i.e., without underlying chronic liver disease). In addition, we aimed to explore the underlying mechanisms and to identify potential therapeutic targets.

## RESULTS

### Hyperammonemia induces liver fibrosis and programmed liver cell death

**Fig. 1.**
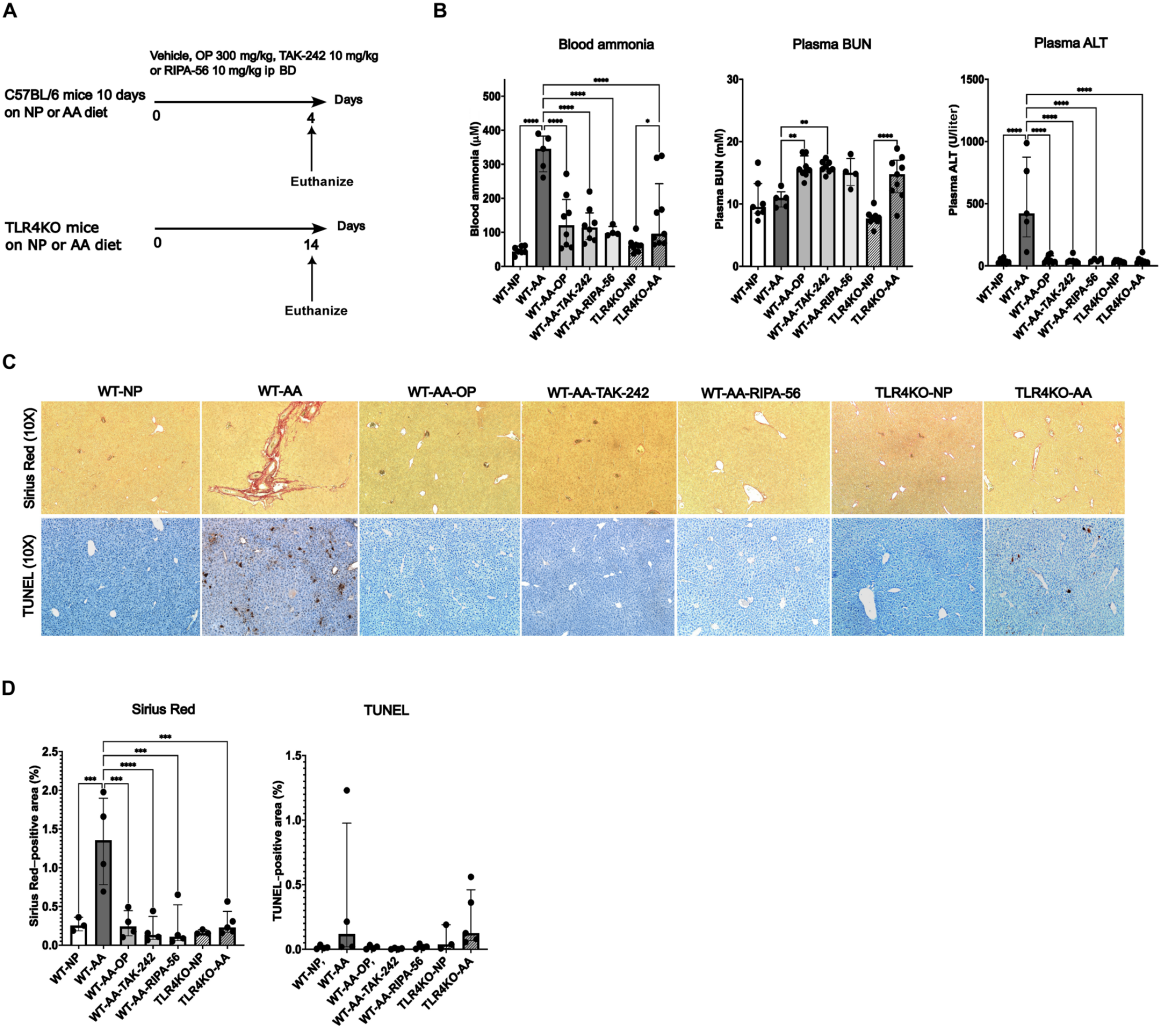
Hyperammonemia induces liver injury, which is prevented by TLR4 and RIPK1 inhibition. (**A**) Experimental design. (**B**) Bar graphs representing circulating ammonia, BUN, and ALT levels. OP, TAK-242, and RIPA-56 treatment significantly reduced circulating ammonia levels. The TLR4KO genotype protected against severe hyperammonemia development. Hyperammonemia did not lead to a significant increase in plasma BUN in WT-AA, as opposed to OP- and TAK-242–treated mice. Hyperammonemia was associated with a significant increase in plasma ALT, which was prevented by OP, TAK-242, and RIPA-56 treatment and by the TLR4KO genotype. (**C**) Bar graphs showing quantification of Sirius Red and TUNEL staining. (**D**) Microscopy imaging of Sirius Red and TUNEL staining. Hyperammonemia was associated with liver fibrogenesis (Sirius Red) and liver cell death (TUNEL). No fibrogenesis or cell death was observed in OP-, TAK-242–, and RIPA-56–treated mice. Data are presented as means ± SD or median ± interquartile range. Groups are compared by ordinary one-way ANOVA with Tukey post hoc test or Kruskal Wallis test with post hoc Dunn’s test. *P < 0.05; **P < 0.01; ***P < 0.001; ****P < 0.0001.

To study the hepatotoxic effects of ammonia, hyperammonemia was induced in healthy wild-type (WT) mice by the addition of an AA mixture to normal powdered (NP) diet in a 1:2 ratio for 14 days (Fig. 1A; for more details, see the Materials and Methods section). The development of hyperammonemia-induced liver injury was explored by plasma alanine aminotransferase (ALT) measurements and liver fibrosis (Sirius Red staining) and cell death [terminal deoxynucleotidyl transferase–mediated deoxyuridine triphosphate nick end labeling (TUNEL) staining] assessment. A significant increase in circulating ammonia levels was observed in WT mice fed with the AA diet (WT-AA) as compared to the control group receiving the NP diet (WT-NP) (333.4 ± 54.5 μM versus 47 ± 11.9 μM, *P* < 0.0001). Treatment of hyperammonemic mice with the ammonia scavenger ornithine phenylacetate (OP; MNK-6106) during the last 4 days of the diet (WT-AA-OP) led to a significant reduction in ammonia levels as compared to WT-AA (333.4 ± 54.5 μM versus 131.6 ± 79.8 μM, *P* < 0.0001) (Fig. 1B). Plasma ALT levels were significantly higher in WT-AA compared with WT-NP (527.2 ± 346.6 versus 35 ± 7.2 U/liter, *P* < 0.0001). The WT-AA-OP group had significantly lower plasma ALT levels compared to WT-AA (527.2 ± 346.6 versus 49.3 ± 10.4 U/liter; Fig. 1B). Hyperammonemia in WT-AA was associated with the development of fibrogenesis (Sirius Red staining) and cell death (TUNEL staining), which was prevented by OP (Fig. 1, C and D).

**Fig. 2.**
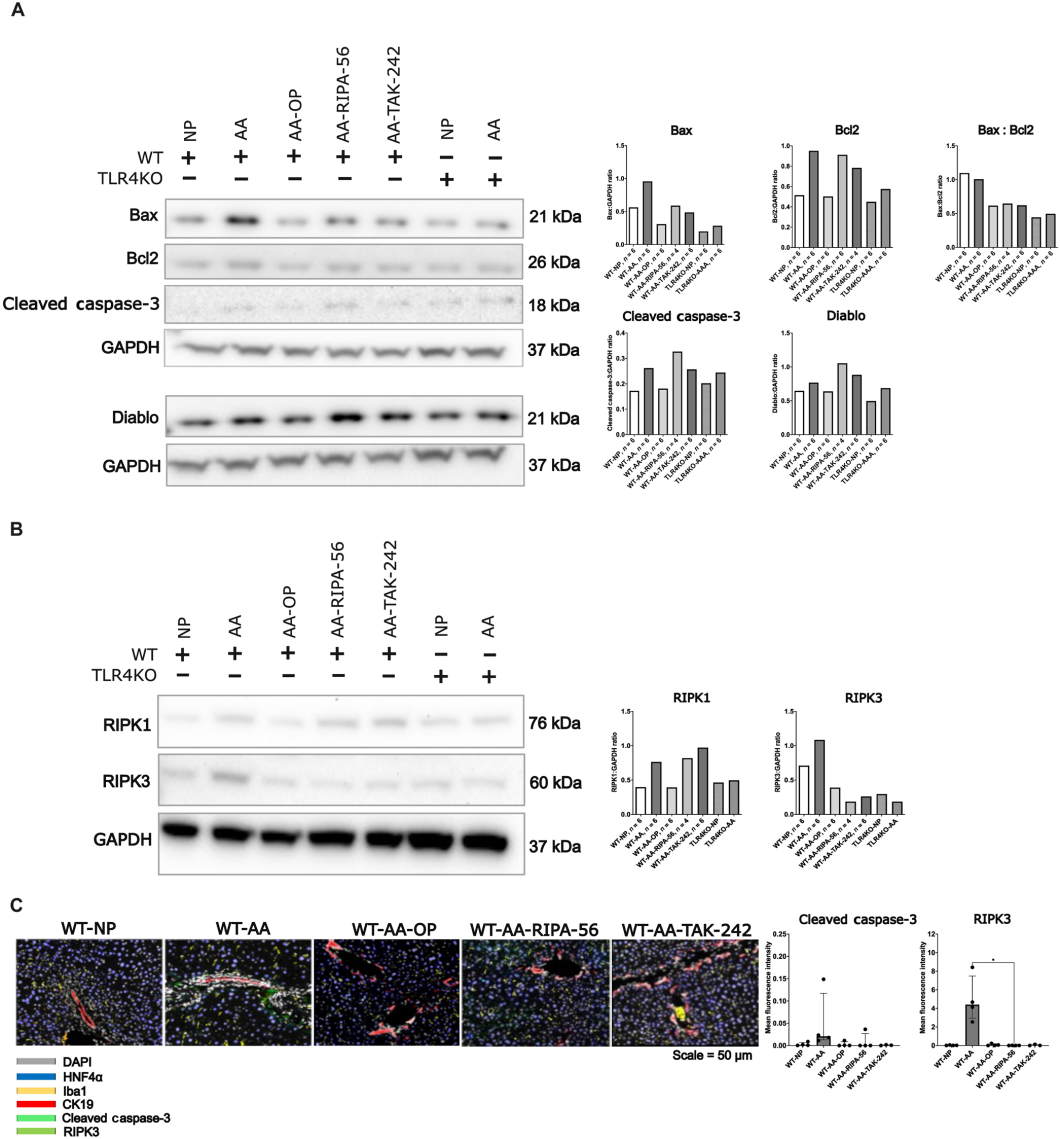
Hyperammonemia induces up-regulation of hepatic apoptotic and necroptotic proteins, which is prevented by TLR4 and RIPK1 inhibition. (**A**) Western blots and quantification of bax, bcl-2, diablo, and cleaved caspase-3 proteins in liver tissue of WT and TLR4KO mice fed with the NP or AA diet with or without treatment with OP, TAK-242, or RIPA-56. Proteins were selected based on a Proteome Profiler Mouse Apoptosis Array (fig. S1) and normalized to glyceraldehyde-3-phosphate dehydrogenase (GAPDH). Hyperammonemia induced up-regulation of bax, bcl2, and cleaved caspase-3. This was reduced in mice treated with OP, TAK-242, or RIPA-56. Up-regulation of these proteins was less pronounced in TLR4KO mice. (**B**) Western blots and quantification of liver RIPK1 and RIPK3 proteins. Hyperammonemia induced up-regulation of both RIPK1 and RIPK3. The up-regulation of RIPK1 was prevented by OP and the up-regulation of RIPK3 by OP, TAK-242, and RIPA-56 treatment. In TLR4KO mice, no changes in RIPK1/3 expression were observed with the AA diet. (**C**) Liver multiplex immunofluorescence analysis of RIPK3 and cleaved caspase-3. The results validate the up-regulation of both proteins in livers of mice fed with the AA diet but not in those treated with OP, TAK-242, or RIPA-56. Western blot analysis was performed using pooled samples (*n* = 4 to 6 animals per group), and therefore, no SDs or statistical comparisons between groups are shown. For multiplex immunofluorescence quantification, groups were compared by ordinary one-way ANOVA with Tukey post hoc test or Kruskal Wallis test with post hoc Dunn’s test. Data are presented as means ± SD or median ± interquartile range. *P < 0.05

To further characterize the mode of liver cell death, a Proteome Profiler Mouse Apoptosis Array (R&D Systems, ARY031) was performed. This assay detects 21 mouse apoptosis-related proteins simultaneously. The main finding of this assay was an up-regulation of proteins involved in the intrinsic apoptosis pathway (p53, bcl-2, bad, and diablo; fig. S1), which was prevented by treatment with OP. Western blots for hepatic bax, bcl-2, diablo, and cleaved caspase-3 proteins confirmed the activation of apoptosis related proteins during hyperammonemia (Fig. 2A). Likewise, key necroptosis proteins receptor-interacting serine/threonine-protein kinase 1 (RIPK1) and RIPK3 showed increased hepatic expression in WT-AA as compared to WT-NP and down-regulation after ammonia lowering by OP (Fig. 2B). These observations were confirmed by a liver multiplex immunofluorescence analysis, which showed that the mean fluorescence intensity of both cleaved caspase-3 and RIPK3 was up-regulated in WT-AA but not in WT-AA-OP, although changes were not statistically significant. Hyperammonemia-induced liver injury in WT-AA was not associated with recruitment of Iba1-positive macrophages or ductular reaction (CK19 staining) (Fig. 2C).

### Inhibition of TLR4 and RIPK1 protects against ammonia-induced liver cell death and severity of hyperammonemia

On the basis of the finding that hyperammonemia induces increased expression of the necroptosis proteins RIPK1 and RIPK3, we aimed at assessing whether inhibition of this pathway would protect against hyperammonemia-induced liver injury and further hyperammonemia. This was achieved by using the selective RIPK1 inhibitor RIPA-56 (Sigma-Aldrich, UK, SML2100) and by applying the AA diet in a RIPK3KO and Casp8^Δhepa^ mouse model. In addition, we chose to explore the protective effect of inhibiting the Toll-like receptor 4 (TLR4) pathway for two reasons: (i) TLR4 is an inducer of RIPK1-independent necroptosis (*23*) and (ii) hyperammonemia has been reported to activate TLR4 and to induce TLR4 up-regulation in brain endothelial cells (*24*). The effect of TLR4 inhibition was studied by either pharmacological treatment with the TLR4 inhibitor TAK-242 (Akaza, UK, lot number M342-017) or by the use of the AA diet in TLR4KO mice (the Jackson Laboratory, B6.B10ScN-*Tlr4^lps-del^*/JthJ, stock number 007227). In WT mice fed with the AA diet, treatment with both the TLR4 inhibitor TAK-242 (WT-AA-TAK-242) and the RIPK1 inhibitor RIPA-56 (WT-AA-RIPA-56) led to a significant reduction in plasma ALT as compared to WT-AA (WT-AA versus WT-AA-TAK-242: 527.2 ± 346.6 versus 41.13 ± 26.8 U/liter; WT-AA versus WT-AA-RIPA-56: 527.2 ± 346.6 versus 45 ± 11.6 U/liter, both *P* < 0.0001; Fig. 1B). No fibrogenesis or TUNEL-positive cells were observed in mice treated with TAK-242 or RIPA-56 (Fig. 1, C and D). TAK-242 and RIPA-56 led to reduced hepatic protein expression of bax as compared to WT-AA but not of the other assessed proteins of the intrinsic apoptosis pathway (Fig. 2A). TAK-242 and RIPA-56 treatment led to a profound decrease in hepatic RIPK3 expression as compared to WT-AA but not of RIPK1 (Fig. 2B). Along with reduced liver injury, blood ammonia levels were significantly lower in WT-AA-TAK-242 and WT-AA-RIPA-56 as compared to WT-AA (WT-AA versus WT-AA-TAK-242: 333.4 ± 54.5 versus 121.1 ± 51.5 μM; WT-AA versus WT-AA-RIPA-56: 333.4 ± 54.5 versus 102.0 ± 14.9 μM, both *P* < 0.0001; Fig. 1B).

Both RIPK3KO and Casp8^Δhepa^/RIPK3KO mice fed with the AA diet (RIPK3KO-AA and Casp8^Δhepa^/RIPK3KO-AA) had lower blood ammonia levels as compared to WT-AA, although this was statistically insignificant. Blood ammonia and plasma ALT levels of the Casp8^Δhepa^-AA group were comparable to those of the WT-AA group. No fibrogenesis (Sirius Red) or cell death (TUNEL staining) was observed in the different experimental groups of this model (fig. S2).

TLR4KO mice fed with the AA diet (TLR4KO-AA) had significantly lower blood ammonia levels compared to WT-AA (149.9 ± 104.6 versus 333.4 ± 54.5 mM, *P* < 0.0001; Fig. 1B). This was associated with a significantly lower plasma ALT (41.2 ± 27.7 versus 527.2 ± 346.6 U/liter, *P* < 0.0001; Fig. 1B) and less fibrosis (Fig. 1, C and D). There was no change in the percentage of TUNEL-positive areas as compared to WT-AA [0.1% (0.1 to 0.5) versus 0.1% (0.02 to 1.0), *P* = 1]. Protein expression levels of bax and bcl-2 were lower in TLR4KO-AA compared with WT-AA but not of diablo and cleaved caspase-3 (Fig. 2A). In TLR4KO mice, the AA diet did not result in increased hepatic protein expression of RIPK1 and RIPK3 (Fig. 2B).

### Hyperammonemia is associated with dysregulation of the urea cycle

#### Plasma urea levels

The urea cycle, located in the periportal hepatocytes, is the main metabolic pathway for ammonia removal and converts ammonia into urea via five enzymatic reactions. We observed that hyperammonemia in the WT-AA mice was not associated with an increase in plasma blood urea nitrogen (BUN) compared to WT-NP (10.8 ± 1.4 versus 10.6 ± 3.2 mM, *P* = 1), whereas treatment with OP and TAK-242 resulted in a significant increase in plasma BUN compared with WT-AA (WT-AA versus WT-AA-OP: 10.8 ± 1.4 versus 15.8 ± 1.7 mM, *P* = 0.004; WT-AA versus WT-AA-TAK-242: 10.8 ± 1.4 versus 15.9 ± 0.9 mM, *P* = 0.007; Fig. 1B). RIPA-56 treatment also led to an increase in plasma BUN compared to WT-AA, although it was not statistically significant (10.8 ± 1.4 versus 15.1 ± 2.3 mM, *P* = 0.1; Fig. 1B). In contrast to the WT model, the AA diet induced a significant rise in plasma BUN levels in TLR4KO mice (TLR4KO-NP: 7.71 ± 1.2 mM versus TLR4KO-AA: 14.3 ± 3.4 mM, *P* < 0.0001; Fig. 1B). These data suggest impaired ureagenesis in hyperammonemia, which was prevented by TLR4 and RIPK1 inhibition.

#### Liver metabolomics

**Fig. 3.**
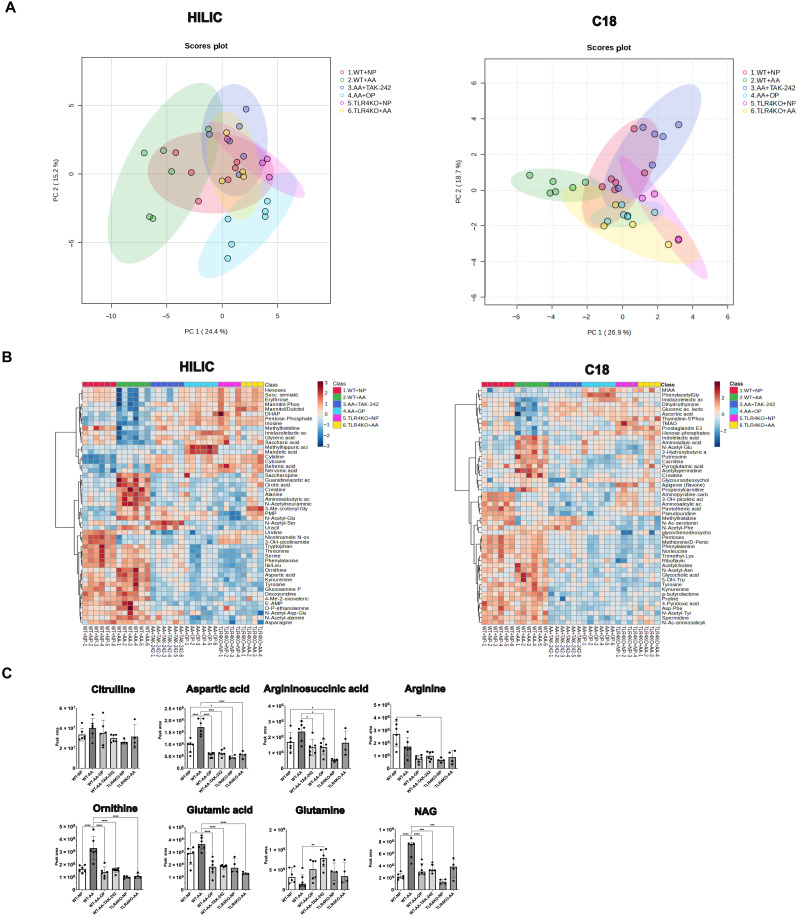
Hyperammonemia induces accumulation of urea cycle metabolites, which is prevented by OP and TLR4 inhibition. (**A**) PCA of the liver metabolomics dataset. (**B**) Heatmaps showing the top 50 differential metabolites in the liver metabolomics analysis, according to ANOVA *P* values < 0.05 between WT-AA and WT-NP. (**C**) Individual peak areas of urea cycle intermediates and related metabolites that were among the top 50 differential metabolites presented in (B). Hyperammonemia in WT-AA was associated with increased peak areas of urea cycle metabolites, primarily ornithine and aspartic acid. In WT-AA, significantly increased peak areas were also observed for glutamic acid and NAG, the allosteric activator of CPS1. These changes were not observed in mice treated with TAK-242 or OP and in TLR4KO mice. Data in (C) are presented as means ± SD. Groups are compared by ordinary one-way ANOVA with Tukey post hoc test. *P < 0.05; **P < 0.01; ***P < 0.001; ****P < 0.0001.

**Fig. 4.**
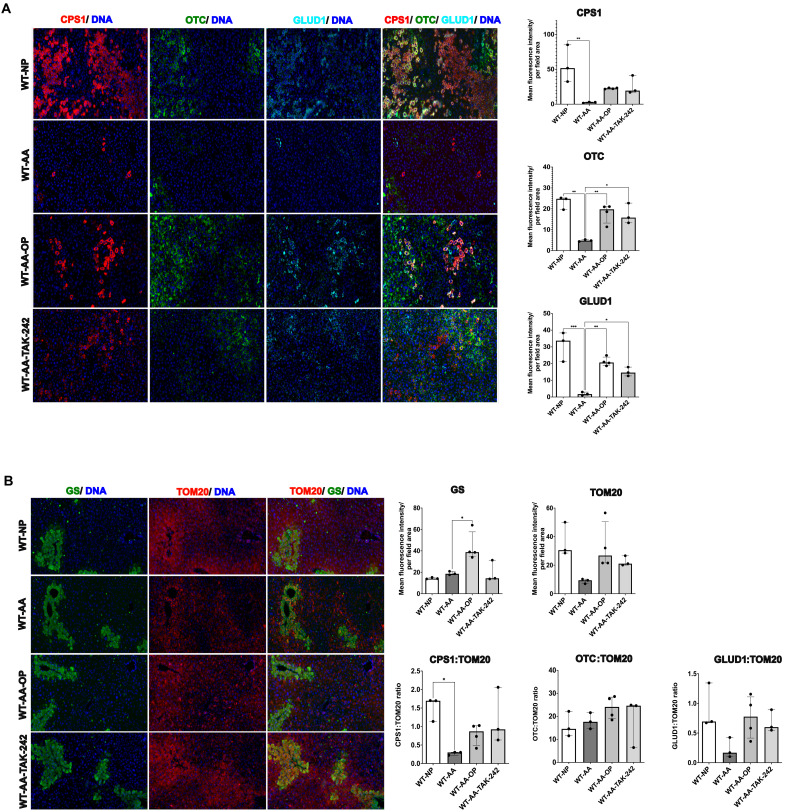
Hyperammonemia induces down-regulation of mitochondrial urea cycle enzymes (UCEs), which is prevented by TLR4 inhibition. (**A**) Liver multiplex immunofluorescence images and quantification of mitochondrial UCEs CPS1, OTC, and GLUD1. Significant weaker signals were seen for all three enzymes in WT-AA mice. A protective effect of OP and TAK-242 treatment was observed. (**B**) Liver multiplex immunofluorescence images and quantification of GS and the mitochondrial marker TOM20. A trend toward a weaker signal of TOM20 was seen in WT-AA as compared to WT-NP, which was increased by OP and TAK-242 treatment. Similar trends in expression of CPS1, OTC, and GLUD1 were observed when they were normalized to TOM20, suggesting that the changes in these enzymes are possibly linked with changes in this mitochondrial marker. Data are presented as means ± SD. Groups are compared by ordinary one-way ANOVA with Tukey post hoc test. *P < 0.05; **P < 0.01; ***P < 0.001.

To further characterize the impact of hyperammonemia on hepatic metabolism, metabolomics analysis of liver tissue was performed in both the WT and TLR4KO mouse models. Untargeted metabolomics experiments were performed by liquid chromatography coupled to high-resolution mass spectrometry (LC-HRMS), using a combination of two complementary chromatographic methods consisting of reversed-phase chromatography (C18 chromatographic column) and hydrophilic interaction chromatography (HILIC) for the analysis of hydrophobic and polar metabolites, respectively (see the Materials and Methods section). Using our internal spectral database, up to 307 distinct metabolites were annotated under these conditions with 219 and 107 metabolites annotated using the HILIC- and C18-based LC-HRMS workflows, respectively (19 metabolites were in common). A full list of the annotated metabolites can be found in table S2. HILIC and C18 datasets can be found in tables S3 and S4, respectively. Principal components analysis (PCA; unsupervised learning) was performed for both databases separately (Fig. 3A). The PCA plot showed clear separation of the WT-AA group, whereas there was considerable overlap among WT-NP, treatment groups, and TLR4KO mice (Fig. 4A). The two principal components (PCs) accounted for 39.6% of total variance (PC1: 24.4% and PC2: 15.2%) for the HILIC data and for 43.5% of total variance (PC1: 26.9% and PC2: 18.7%) for the C18 data.

The top 50 metabolites that were most significantly affected by hyperammonemia in WT-AA versus WT-NP [according to analysis of variance (ANOVA) *P* values < 0.05] were first highlighted for the HILIC and C18 data through heatmap representations (Fig. 3B). These metabolites primarily included intermediates of the urea cycle and pathways that are interrelated with the urea cycle, such as the polyamine, pyrimidine and purine metabolic pathways (fig. S4). In addition, these metabolites included intermediates of the Krebs cycle (primarily an increase in hepatic malic acid) and the mitochondrial β-oxidation pathway (primarily an increase in metabolites involved in the carnitine shuttle). These results are described and discussed in more detail in the Supplementary Results section.

Regarding the urea cycle, accumulation of its intermediates was observed in the setting of hyperammonemia. Apart from arginine, all cytosolic intermediates and nitrogen donors of the urea cycle were increased in WT-AA compared with WT-NP. This was most pronounced for ornithine (*P* < 0.0001) and aspartic acid (*P* < 0.0001) (Fig. 3C). This is in accordance with the results of blood AA profiling, which are further described in the Supplementary Results section and fig. S3. In OP- and TAK-242–treated mice, levels of ornithine and aspartic acid were reduced significantly and comparable to those in WT-NP (both *P* < 0.0001). The obligatory allosteric activator of the rate-limiting UCE carbamoyl phosphate synthetase 1 (CPS1), hepatic *N*-acetylglutamic acid (NAG), was significantly increased in WT-AA compared with WT-NP (*P* < 0.0001; Fig. 3C). In OP- and TAK-242–treated mice, NAG levels were significantly reduced compared with WT-AA (both *P* < 0.001). Glutamic acid, which is generated by the action of liver-type glutaminase on glutamine in the mitochondria of periportal cells, was increased in WT-AA as compared to WT-NP (*P* = 0.049; Fig. 3C). OP and TAK-242 treatment significantly reduced hepatic glutamic acid levels compared to WT-AA (*P* < 0.0001 and *P* < 0.0001, respectively).

In the TLR4KO model, no diet-induced changes in glutamic acid and glutamine levels were observed. Also, there were no significant changes in the urea cycle intermediates between TLR4KO-NP and TLR4KO-AA (Fig. 3C). When looking at the effect of the genotype on substrate utilization, the TLR4KO animals appeared to rely more on carbohydrates compared to the WT-AA animals; a significant decrease in carbohydrates and intermediates of glycolysis was observed in the WT-AA but not in the TLR4KO-AA animals (Fig. 3B). This could partially explain the observed lower levels of blood ammonia in the TLR4KO-AA mice compared with WT-AA.

#### Protein expression of UCEs

To determine the possible mechanisms of the findings from the liver metabolomics analysis, liver multiplex immunofluorescence was performed to assess the expression of the key UCEs CPS1 and ornithine transcarbamylase (OTC), both located in the mitochondria. Mean fluorescence intensity per field area of CPS1 and OTC was profoundly weaker in WT-AA compared to WT-NP (CPS1: *P* = 0.0052; OTC: *P* = 0.001) (Fig. 4A). For both CPS1 and OTC, OP and TAK-242 treatment led to a stronger signal compared to WT-AA, which was most pronounced for OTC (OP: *P* = 0.0064; TAK-242: *P* = 0.0134) (Fig. 4A). The same pattern was observed for glutamate dehydrogenase (GLUD1), a mitochondrial enzyme that converts glutamic acid to α-ketoglutaric acid. This reaction yields ammonia as a substrate for CPS1 to drive the urea cycle. OP and TAK-242 treatment significantly increased the mean fluorescence intensity per field area of GLUD1 as compared to WT-AA (*P* = 0.0019 and *P* = 0.03, respectively; Fig. 4A).

The signal of GS, the pericentral zonation marker (and key enzyme in the metabolism of ammonia that bypasses the urea cycle), was similar between WT-AA and WT-NP. As expected, GS was profoundly increased in WT-AA-OP as compared to the other groups as this drug is known to induce increased expression and activity of GS (*P* < 0.05) (Fig. 4B) (*25*). There was a trend toward a weaker signal of the mitochondrial marker TOM20 in WT-AA as compared to WT-NP, although this was not statistically significant. Both TAK-242 and OP led to an increase in the TOM20 signal (Fig. 4B). Similar trends in expression of CPS1, OTC, and GLUD1 were observed when they were normalized to TOM20 expression, suggesting that the changes in these enzymes are possibly linked with changes in this mitochondrial marker.

### TLR4 inhibition protects against hyperammonemia in clinically relevant animal models

To validate the finding that TLR4 inhibition protects against hyperammonemia, we studied the effect of the TLR4 antagonist TAK-242 in two clinically relevant animal models of hyperammonemia.

#### Bile duct–ligated rats

**Fig. 5.**
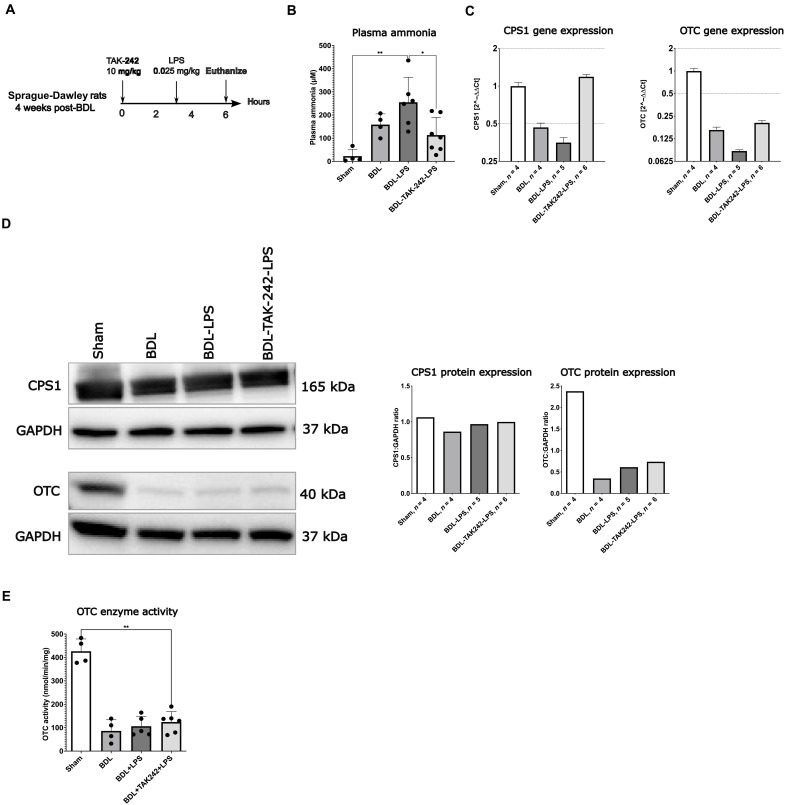
TLR4 inhibition reduces circulating ammonia levels and preserves gene expression of urea cycle enzymes (UCEs). (**A**) Study design. (**B**) A stepwise increase in plasma ammonia was observed throughout the sham, BDL, and BDL-LPS group. Prophylactic TAK-242 treatment resulted in significantly lower ammonia levels compared to BDL-LPS. (**C**) Bar graphs representing hepatic gene expression (2^−ΔΔCt^) of the key UCEs CPS1 and OTC. In BDL and BDL-LPS groups, significantly lower gene expression levels of CPS1 and OTC were observed as compared to sham. Prophylactic TAK-242 treatment restored gene expression levels of CPS1 but not of OTC. (**D**) Western blots and quantification of liver CPS1 and OTC. No changes in CPS1 protein expression levels were observed between groups. A significant decrease in OTC protein expression was observed in the BDL group as compared with sham. No impact of LPS on OTC protein expression was observed. (**E**) In line with Western blot analysis, OTC enzyme activity was significantly reduced in BDL animals. No effect of LPS on OTC enzyme activity was observed. Data are presented as means ± SD. For (B), (C), and (E), groups are compared by ordinary one-way ANOVA with Tukey post hoc test. Western blot analysis was performed using pooled samples (*n* = 4 to 6 animals per group), and therefore, no SDs or statistical comparisons between groups are shown. *P < 0.05; **P < 0.01.

This rat model has been described in more detail previously (see also the Materials and Methods section) (*26*). Sprague-Dawley rats were studied 4 weeks after sham or bile duct ligation (BDL) surgery. One group received a second hit with an intraperitoneal injection of lipopolysaccharide (LPS; 0.025 mg/kg) (*Klebsiella pneumoniae*, Sigma-Aldrich, UK). TAK-242 [10 mg/kg intraperitoneally (ip)] was administered prophylactically 3 hours prior to the LPS injection. In that study, we have described that LPS administration in BDL rats induces liver and multiorgan failure with predominantly necroptotic hepatocyte cell death. The TLR4 pathway was shown to be an important mediator of hepatocyte cell death, and TAK-242 protected against LPS-induced liver cell death. In the present study, we aimed at evaluating the impact of BDL, LPS, and TAK-242 treatment on ammonia levels and urea cycle function. A stepwise increase in plasma ammonia levels was observed in the sham, BDL, and BDL-LPS groups (sham: 22.5 ± 29.9 μM, BDL: 158.6 ± 46.3 μM, BDL-LPS: 255.0 ± 107.6 μM; Fig. 5B). BDL-LPS rats pretreated with TAK-242 had significantly lower plasma ammonia levels as compared to those in the BDL-LPS group (113.2 ± 76.01 versus 255.0 ± 107.6 μM, *P* = 0.02) and had a lower coma rate (85% versus 0%).

To assess urea cycle function, hepatic gene and protein expression of CPS1 and OTC, as well as OTC enzyme activity was assessed. We observed a significant decrease in hepatic gene expression of CPS1 and OTC in the BDL group as compared to sham (Fig. 5C). This was even more pronounced in the BDL-LPS group. Prophylactic TAK-242 treatment restored CPS1 gene expression to a level comparable with sham animals. Gene expression levels of OTC were nonsignificantly increased in BDL-TAK-242-LPS as compared with the BDL-LPS group. On protein level, no changes in CPS1 expression were observed (Fig. 5D). For OTC protein expression, a marked reduction was observed in BDL rats as compared to sham. No impact of LPS on OTC protein expression was observed. In line with this, OTC enzyme activity was significantly reduced in BDL rats as compared to sham, but no additional effect of LPS was observed (Fig. 5E). The discrepancy between the effect of LPS on gene and protein level might be related to the fact that rats were euthanized 3 hours post-LPS injection, which may be too short to observe an effect on protein level.

#### OTC^spf-ash^ mice

**Fig. 6.**
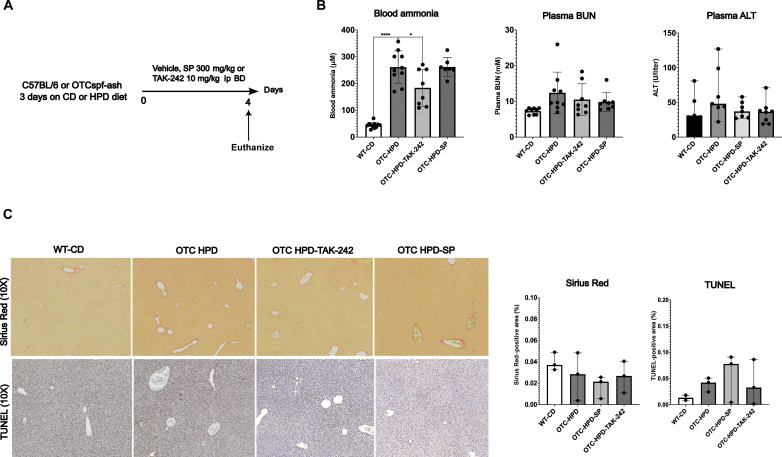
TLR4 inhibition reduces circulating ammonia levels in rodent models of genetic urea cycle disorder. (**A**) Study design. (**B**) Bar graphs representing circulating ammonia, BUN, and ALT levels in the OTC^spf-ash^ mouse model. Data show a significant increase in blood ammonia in OTC-HPD mice compared with WT-CD, which was prevented by TAK-242 treatment but not by SP. No significant changes in plasma ALT and BUN were observed among the groups. (**C**) Microscopy and quantification of Sirius Red and TUNEL staining. No fibrogenesis or cell death was observed in this model. Data are presented as means with SD or median with interquartile range. Groups are compared by ordinary one-way ANOVA with Tukey post hoc test or Kruskal Wallis test with post hoc Dunn’s test. *P < 0.05; ****P < 0.0001.

A model of UCD-induced hyperammonemia was studied. OTC deficiency, an X-linked genetic disease, is the most common type of UCDs in human (*27*). We studied male (25 ± 5 g) OTC^spf-ash^ mice (the Jackson Laboratory, B6EiC3Sn *a*/*A*-*Otc^spf-ash^*/J, stock number 001811). Hyperammonemia was induced by a high-protein diet (HPD) for the duration of 7 days (please see the Materials and Methods section). During the last 4 days of the model (i.e., days 4 to 7), mice were treated with either TAK-242 (10 mg/kg ip) twice daily or sodium phenylacetate (SP; 300 mg/kg per day, mixed with feed). SP is the standard of care in the treatment of patients with OTC deficiency. OTC-HPD mice had significantly higher circulating ammonia levels compared to WT mice fed with a control diet (WT-CD) (261.3 ± 60.5 μM versus 43.9 ± 11.0 μM, *P* < 0.0001). Treatment with TAK-242 led to significantly lower circulating ammonia levels compared to OTC-HPD groups (183.9 ± 68.5 versus 261.3 ± 60.5 μM, *P* = 0.035). Treatment with SP did not reduce blood ammonia levels compared to OTC-HPD (Fig. 6B). No significant changes in plasma BUN were observed among groups, although there was a trend toward increased plasma BUN in OTC-HPD as compared to WT-CD [9.7 (8.7 to 14.6) versus 7.6 (6.3 to 8.1) mM, *P* = 0.08). No significant changes in plasma ALT, fibrogenesis (Sirius Red), and cell death (TUNEL staining) were observed among groups (Fig. 6C).

To further characterize and validate the impact of hyperammonemia on hepatic metabolism, we extended the liver metabolomics analysis to this mouse model. Data on HILIC and C18 datasets can be found in tables S5 and S6, respectively. PCA was performed for both databases separately (fig. S6A). The PCA plots showed that OTC^spf-ash^ mice have a distinct metabolic profile from WT and TLR4KO mice, but there was considerable overlap among the groups within the OTC^spf-ash^ model. The top 50 metabolites that were most differentially affected by hyperammonemia are presented in the heatmaps in fig. S6. No marked changes were observed between the experimental groups in the OTC^spf-ash^ mouse model.

### Ammonium chloride does not directly induce hepatocyte cell death and TLR4 transactivation in vitro

To better understand the mechanism of hyperammonemia-induced liver injury as observed in the in vivo rodent models, in vitro studies using the human hepatocyte cell line 5 (HHL5) cells and HEK-Blue hTLR4 reporter cells were performed. The stimulation of HHL5 cells with clinically relevant concentrations of NH_4_Cl did not impact on cell viability [adenosine triphosphate (ATP) assay] and did not induce cell death [cell death enzyme-linked immunosorbent assay (ELISA)] (fig. S7). Similarly, stimulation of HEK-Blue hTLR4 reporter cells with a wide range of NH_4_Cl concentrations did not induce TLR4 transactivation (fig. S7). These data point toward an indirect effect of hyperammonemia on both hepatocyte injury and TLR4 transactivation.

### Ammonium chloride reduces oxygen consumption rate in primary mouse hepatocytes

**Fig. 7.**
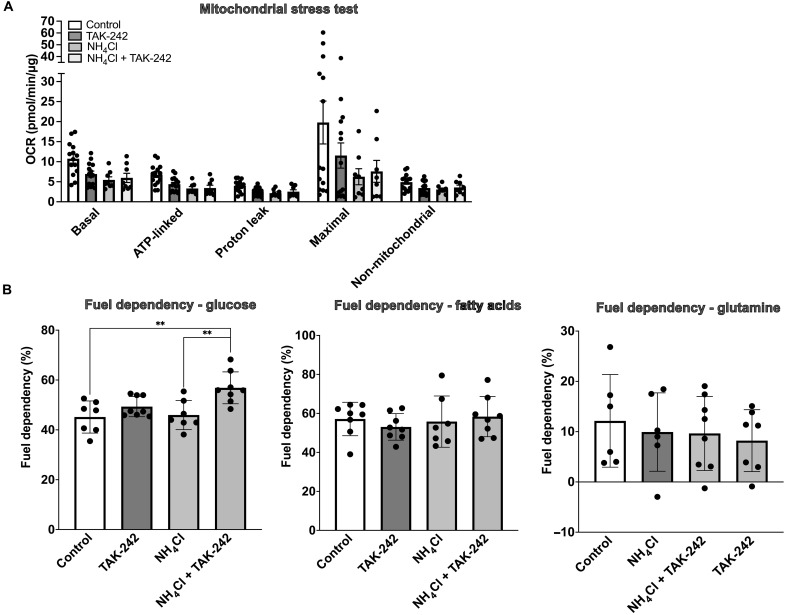
Ammonium chloride reduces the oxygen consumption rate (OCR) in primary mouse hepatocytes (PMHs). (**A**) Bar chart showing mitochondrial respiration changes measured by the Seahorse flux analyzer in PMHs exposed to 5 mM NH_4_Cl with or without 0.2 μM TAK-242 or TAK-242 alone. The OCR was analyzed for basal respiration, ATP-linked respiration, proton leak respiration, maximal respiration, and nonmitochondrial oxygen consumption. The results show a nonsignificant decrease in OCR of PMHs upon exposure to NH_4_Cl. TAK-242 treatment led to a trend toward increased OCR for basal and maximal respiration. (**B**) Bar charts representing the results of the Seahorse XF Mito Fuel Flex test, showing an increase in glucose-dependent ATP generation in PMHs exposed to NH_4_Cl + TAK-242. No changes in fatty acids or glutamine-dependent ATP generation were observed among the groups. Groups are compared by ordinary one-way ANOVA with Tukey post hoc test. **P < 0.01.

To assess whether hyperammonemia and TAK-242 treatment affect energy metabolism of hepatocytes, measurements of oxygen consumption rate (OCR) were performed in primary mouse hepatocytes (PMHs) that were exposed to NH_4_Cl with or without treatment with 0.2 μM TAK-242. In addition, dependency of mitochondrial respiration on the three main sources feeding the tricarboxylic acid cycle (i.e., glucose, glutamine, and fatty acids) was determined. On the basis of the findings in the HHL5 cells in which no effect on cell viability was observed with clinically relevant NH_4_Cl concentrations, substantially higher dosages of NH_4_Cl were used in this experiment (i.e., 2 to 10 mM). Microscopically, no cell death was observed. A dose-dependent decrease in OCR was observed in PMHs that were incubated with NH_4_Cl as compared to controls (fig. S8). A dose of 5 mM NH_4_Cl was used for further experiments. TAK-242 treatment of NH_4_Cl-exposed PMHs resulted in a tendency toward improved mitochondrial function as reflected by an increase in OCR for basal and maximal respiration as compared to untreated cells. This was accompanied by an increase in glucose-dependent ATP generation. No changes were observed for fatty acids and glutamine-dependent ATP generation upon exposure to 5 mM NH_4_Cl with or without TAK-242 (Fig. 7).

## DISCUSSION

The results of this study show that hyperammonemia induces liver fibrosis and programmed liver cell death (apoptosis and necroptosis), which is associated with dysregulation of the urea cycle as reflected by down-regulation of key UCEs, accumulation of urea cycle intermediates, and impaired ureagenesis. This may lead to a vicious cycle in which hyperammonemia leads to a further increase in ammonia, which culminates in further liver injury. Targeting of the key necroptosis regulator RIPK1 or TLR4, which is known to be an inducer of the necroptosis pathway upstream of RIPK1, protects against hyperammonemia-induced liver injury and reduces circulating ammonia levels. Therefore, these represent previously unidentified therapeutic targets for the treatment of hyperammonemia, which was confirmed in animal models of UCD and liver cirrhosis, which are both clinically relevant.

The potential role of hyperammonemia as a hepatotoxin and a potential contributor to liver disease progression is supported by previous studies. Ammonia has been shown to drive the progression of metabolic dysfunction–associated fatty liver disease in an animal model by promoting hepatic fibrogenesis (*17*). The mechanism underlying this deleterious effect of ammonia was shown to be mediated through its effect on HSC activation (*28*). Furthermore, histopathological studies in patients with UCD have shown evidence of progressive fibrosis and cirrhosis development, suggesting that ammonia itself may drive hepatotoxicity (*18*, *29*). In the present study, we confirm that chronic hyperammonemia that is induced by an AA diet promotes fibrogenesis and induces liver cell death (apoptosis and necroptosis), which was prevented by the ammonia-lowering drug OP.

Necroptosis is a programmed form of immunogenic cell death. It uses the same upstream signaling cascade as apoptosis, but the result is cellular swelling and leakage. Whether a cell goes into apoptosis or necroptosis relies on two RIPKs: RIPK1 and RIPK3. Activation of caspase-8 promotes apoptosis by cleaving RIPK1/3, whereas inhibition of caspase-8 promotes assembly of “the necrosome” RIPK1/3. Activated RIPK3 subsequently recruits and phosphorylates mixed lineage kinase domain-like protein (MLKL). Activated MLKL oligomerizes and translocates to the plasma membrane, inducing membrane permeabilization. The cells then release extracellular vesicles and danger-associated molecular patterns (DAMPs) that can trigger inflammatory cascades resulting in systemic inflammation (*30*). Necroptosis has been shown to play a potentially vital role in different stages of acute and chronic liver diseases (*31*). In acute-on-chronic liver failure (ACLF), histological studies have shown marked evidence of necroptosis in rodent models and patients (*25*, *32*). In the present study, hyperammonemia was associated with increased hepatic expression of both RIPK1 and RIPK3. Inhibition of RIPK1 with its selective inhibitor RIPA-56 protected against hyperammonemia and restored RIPK3 protein expression levels and prevented liver cell death and fibrogenesis. RIPK3KO and Casp8^Δhepa^/RIPK3KO mice fed with the AA diet had slightly lower circulating ammonia levels as compared to WT animals, whereas ammonia levels in Casp8^Δhepa^ mice were similar to those of the WT-AA group. This suggests that RIPK3-dependent necroptosis, as opposed to caspase-8–dependent apoptosis, may be the primary form of cell death induced by hyperammonemia. However, an important limitation of the results of this second batch of the AA diet mouse model is the fact that the control group (i.e., WT-NP) had relatively high ammonia levels and there was no significant increase in blood ammonia levels observed in WT-AA as compared to WT-NP. This may also explain the lack of increased plasma ALT levels and the absence of fibrogenesis and cell death in the WT-AA group. This experiment was performed in a different lab than the WT and TLR4KO mice due to the access to the RIPK3KO, Casp8^Δhepa^, and Casp8^Δhepa^/RIPK3KO mice. Although the genetic background of control groups, body weights, dietary intervention, and housing conditions were identical, these discrepancies may potentially be explained by differences in consumption of the AA diet, microbiome composition, stress levels, or the method of blood withdrawal. Further studies are therefore needed to validate these data.

In addition to inhibiting RIPK1 and RIPK3, inhibition of the TLR4 pathway by TAK-242 or TLR4 deficiency was found to significantly reduce circulating ammonia levels, which was validated in clinically relevant models of hyperammonemia due to cirrhosis or UCD. The TLR4 pathway is known to promote fibrogenesis via HSCs, Kupffer cells, and liver sinusoidal endothelial cell activation (*33*, *34*). In addition, activation of TLR4 can directly initiate necroptosis by binding to TIR-domain-containing adaptor-inducing beta interferon (TRIF), which has a Receptor interacting protein Homotypic Interaction Motif (RHIM) domain that can bind directly to RIPK1 (*23*, *35*). LPS injection in BDL animals leads to a switch from apoptotic (caspase-3/7–mediated) cell death to predominantly necroptotic (RIPK3-mediated) cell death (*26*). In that study, TAK-242 treatment was shown to significantly reduce LPS-induced necroptosis. In the present study, TAK-242 treatment and knockout of the TLR4 receptor prevented fibrogenesis and cell death and reduced RIPK3 expression levels. We therefore suggest that hyperammonemia leads to liver injury, which is associated with TLR4-mediated liver necroptosis.

The occurrence of fibrogenesis and liver cell death during hyperammonemia could not be validated in the model of OTC^spf-ash^ mice fed with an HPD. This is likely to be explained by the relatively short duration of this model (7 days as opposed to 14 days in the WT model). Because of the fragility of the OTC^spf-ash^ mouse strain and their defective urea cycle, a longer duration of this model using this diet was not possible. However, previous histopathological studies in patients with UCDs have provided evidence of fibrogenesis at older ages (*18*, *36*). Nevertheless, TAK-242 treatment was shown to significantly reduce circulating ammonia levels in this model.

Along with the induction of hyperammonemia in WT mice using the AA diet, we observed an insufficient increase in plasma urea levels. Treatment with OP, TAK-242, and RIPA-56 led to a significant increase in circulating urea levels. In addition, liver metabolomics and blood AA profiling revealed accumulation of key urea cycle metabolites (mainly ornithine and aspartic acid) in livers and in the circulation of hyperammonemic mice. Hepatic protein expression of the UCEs, OTC, CPS1, and GLUD1 were significantly down-regulated in hyperammonemia, which was restored upon treatment with OP and TAK-242. Together, these data suggest impaired ureagenesis during induced hyperammonemia, which is likely to be secondary to hyperammonemia-induced liver injury, that may, in turn, lead to a vicious cycle of further hyperammonemia and further injury. Along with the reduced expression of the key mitochondrial UCEs, we observed decreased hepatic expression of the mitochondrial marker TOM20 in hyperammonemia, indicating a reduction in the mitochondrial number. Recent studies in the brain showed that hyperammonemia induces neuronal mitochondrial dysfunction, cellular senescence, and neuronal cell death (*37*–*39*). We performed further in vitro studies to evaluate whether NH_4_Cl directly induces hepatic mitochondrial dysfunction and cell death.

Studies with HHL5 hepatocytes revealed that clinically relevant concentrations of NH_4_Cl do not directly induce hepatocyte cell death in vitro. These data suggest that hepatocyte cell death in hyperammonemia, as observed in the in vivo models, is likely to be an indirect and hepatocyte-independent effect. The exact mechanism remains to be explored. The observed hyperammonemia-induced fibrogenesis may be mediated through direct activation of HSCs, as we have previously shown that ammonia directly induces deleterious functional and morphological effects on HSCs, both in vitro and in vivo (*17*, *28*). In addition, we observed that incubation of NH_4_Cl with HEK-Blue hTLR4 reporter cells did not lead to TLR4 transactivation. As TLR4 inhibition in vivo was found to protect against hyperammonemia and hyperammonemia-induced liver injury, these data point toward indirect activation of TLR4 during hyperammonemia, for example, through secreted DAMPs. Further unraveling the complex mechanism of hyperammonemia-induced liver injury (especially the identification of involved cell types and sequence of events) will be subject to future studies. Despite the observation that clinically significant concentrations of NH_4_Cl did not induce hepatocyte cell death in vitro, a dose-dependent decrease in mitochondrial respiration was observed in PMHs upon exposure to high concentrations of NH_4_Cl (2 to 10 mM). Even with these high concentrations (which are much higher than observed under disease conditions), no cell death was observed. Concomitant treatment of PMHs with TAK-242 led to a slight improvement of OCR, although not significantly. In addition, TAK-242 treatment of PMHs exposed to NH_4_Cl led to an increase in glucose-dependent ATP production. These data suggest that hyperammonemia induces hepatocyte mitochondrial dysfunction and that TAK-242 may have a protective effect. However, given the very high concentrations of ammonia needed to elicit these changes, its clinical significance remains uncertain.

A recent paper by Mercado-Gómez *et al.* describes another potential link between the TLR4 pathway and ammonia metabolism (*40*). In a rodent model of steatotic liver disease, they found up-regulation of hepatic glutaminase type 1 and TLR4, which was associated with the buildup of hepatic ammonia. Pharmacological inhibition of TLR4 was shown to reduce hepatic glutaminase type 1 expression and hepatic ammonia content. Although we did not explore the role of glutaminase type 1 in the present study, it may partly explain the protective effect of TLR4 in our hyperammonemia models.

In conclusion, the results of this study provide insights into the deleterious effect of hyperammonemia on progression of liver injury and fibrosis, which is associated with mitochondrial dysfunction and appears to be TLR4 dependent, but the exact mechanism of the interaction is not clear. The observation that treatment with a TLR4 antagonist reduces circulating ammonia to levels seen with ammonia scavengers in clinically relevant models of hyperammonemia provides the rationale for proof-of-concept clinical studies with TLR4 antagonists.

## MATERIALS AND METHODS

### Study design

Three distinct rodent models of hyperammonemia were studied. All experimental procedures were performed in compliance with the United Kingdom Animal (Scientific Procedures) Act of 1986 and with the European directive 2010/63/EU, approved by the University College London (UCL) Animal Welfare and Ethical Review Body under full project license (no. 14378) and by the University Hospital RWTH Aachen (no. 84-02.04.2016.A080). Experiments were performed according to the ARRIVE guidelines. The animals were group housed in individually ventilated cages and kept on a 12-hour/12-hour light/dark cycle with ad libitum access to water and food. All experiments were terminated by exsanguination under general anesthesia with isoflurane (2% isoflurane in oxygen, Piramal Healthcare, USA) in the mornings.

#### A mouse model of chronic hyperammonemia in treatment naïve mice

Hyperammonemia was induced in male (25 ± 5 g) WT C57BL/6 mice by the addition of an AA mixture to NP diet in a 1:2 ratio for 14 days. This AA mixture was previously developed as an ammoniagenic diet and mimics the composition of the hemoglobin molecule, which is unique in lacking the essential AA isoleucine (*41*). For the exact diet composition, please see table S1. Dose finding studies revealed that, with this ratio and duration of the diet, circulating ammonia levels between 300 and 400 μM were achieved, which are pathophysiologically relevant concentrations (fig. S5). To validate the ammonia-specific effect on liver injury, one group of mice was treated with the ammonia scavenger OP (MNK-6106) twice daily on days 10 to 14 (300 mg/kg ip).

Two additional interventions were studied: the TLR4 antagonist TAK-242 (Takeda, Japan; Akaza, UK, lot number M342-017) and selective RIPK1 inhibitor RIPA-56 (Sigma-Aldrich, UK, SML2100). Both drugs were administered twice daily on days 10 to 14 at a dose of 10 mg/kg ip. To validate the effects of TLR4 and RIPK1 antagonism in hyperammonemia, the AA diet was also studied in TLR4KO mice (the Jackson Laboratory, B6.B10ScN-*Tlr4^lps-del^*/JthJ, stock number 007227), RIPK3KO mice, Caspase 8^Δhepa^ (Casp8^Δhepa^) mice, and Caspase 8^Δhepa^/RIPK3KO mice.

The following groups were studied:

Batch 1: WT and TLR4KO mice

1) WT + NP diet + vehicle (WT-NP; *n* = 7)

2) WT + AA diet + vehicle (WT-AA; *n* = 5)

3) WT-AA + OP (WT-AA-OP; *n* = 8)

4) WT-AA + TAK-242 (WT-AA-TAK-242; *n* = 8)

5) WT-AA + RIPA-56 (WT-AA-RIPA-56; *n* = 4)

6) TLR4KO + NP diet + vehicle (TLRKO-NP; *n* = 9)

7) TLR4KO + AA diet + vehicle (TLR4KO-AA; *n* = 9)

Batch 2: RIPK3KO, Casp8^Δhepa^, and Casp8^Δhepa^/RIPK3KO mice

1) WT-NP (*n* = 5)

2) WT-AA (*n* = 7)

3) RIPK3KO-AA (*n* = 6)

4) Casp8^Δhepa^-AA (*n* = 7)

5) Casp8^Δhepa^/RIPK3KO-AA (*n* = 7)

#### Hyperammonemia in OTC-deficient mice

To validate our findings in the AA diet–induced chronic hyperammonemia models, we aimed at studying the effect of TLR4 inhibition in a clinically relevant model of UCD. OTC is a key enzyme of the urea cycle, and OTC deficiency, an X-linked genetic disease, is the most common type of UCD in human (*25*). We studied male (25 ± 5 g) OTC^spf-ash^ mice (the Jackson Laboratory, B6EiC3Sn *a*/*A*-*Otc^spf-ash^*/J, stock number 001811). The spf-ash (sparse fur, abnormal skin, and hair) mutation has a unique effect on OTC biogenesis: In single-mutant males, two OTC enzyme precursors are produced, together resulting in 10% of WT precursor levels. They give rise to the residual 5 to 10% hepatic OTC activity in OTC^spf-ash^ mice. Therefore, these mice become quickly hyperammonemic upon factors such as high protein intake, stress, and inflammation (*42*). Hyperammonemia was induced by an HPD for the duration of 7 days. The diet consisted of DietGel 76A (Datesand Group, catalog no. 72-07-5022) to which casein powder (MPBio, catalog no. 901293) was added to achieve a total protein content of 36%. During the last 4 days of the model (i.e., days 4 to 7), mice were treated with either TAK-242 (10 mg/kg ip) twice daily or SP (300 mg/kg per day, mixed with feed). SP is the standard of care in the treatment of patients with OTC deficiency.

The following groups were studied:

1) WT + CD + vehicle (WT-CD; *n* = 11)

2) OTC^spf-ash^ + HPD + vehicle (OTC-HPD; *n* = 10)

3) OTC^spf-ash^ + HPD + TAK-242 (OTC-HPD-TAK-242; *n* = 8)

4) OTC^spf-ash^ + HPD + SP + vehicle (OTC-HPD-SP; *n* = 7)

#### Hyperammonemia in a rodent model of ACLF

This rat model of ACLF has been described in more detail previously (*26*). Briefly, Sprague-Dawley rats (260 ± 20 g, aged 8 to 10 weeks) were studied 4 weeks after sham or BDL surgery. ACLF was induced by the intraperitoneal injection of LPS (0.025 mg/kg; *Klebsiella pneumoniae*, Sigma-Aldrich, UK). TAK-242 (10 mg/kg ip) was administered prophylactically 3 hours prior to the LPS injection.

The following groups were studied:

1) Sham + vehicle + vehicle (Sham; *n* = 4)

2) BDL + vehicle + vehicle (BDL; *n* = 4)

3) BDL + LPS + vehicle (BDL-LPS; *n* = 6)

4) BDL + TAK-242 + LPS (BDL-TAK-242-LPS; *n* = 7)

### Drug preparation

Drug preparation of TAK-242 was performed as described previously (*26*). Briefly, TAK-242 was dissolved in *N*-methyl-2-pyrrolidone (NMP; Fisher Scientific, UK) at a concentration of 200 mg/ml. This stock solution was further diluted to 40 mg/ml with NMP. The final working solution of 1 mg/ml was prepared using 30% (2-hydroxypropyl) β-cyclodextrin (Sigma-Aldrich, catalog number H107)/0.5% citric acid. The vehicle control solution was prepared by adding NMP to 30% (2-hydroxypropyl) β-cyclodextrin/0.5% citric acid (1:40 ratio). RIPA-56 was prepared similarly to TAK-242 by diluting it to a final working solution of 0.3 mg/ml with NMP and 30% (2-hydroxypropyl) β-cyclodextrin. OP was dissolved in sterile phosphate-buffered saline at a concentration of 300 mg/kg. SP was mixed with the HPD at a concentration of 300 mg/kg per day.

### Sampling and storage of blood and tissues

Arterial blood samples were taken from the left ventricle of the heart at time of experiment termination by exsanguination under general anesthesia as described previously. Immediately following blood collection in an EDTA tube on ice, whole blood ammonia was measured using a portable ammonia analyzer (DRI-CHEM NX10N, FujiFilm Co., Saitama, Japan). The remaining plasma was stored at −80°C. Part of the liver tissue was snap frozen in liquid nitrogen and stored at −80°C, and part was preserved in 10% neutral buffered formalin for 24 hours prior to embedding in paraffin.

### Blood biochemistry

Plasma BUN and ALT levels were measured using an automatic biochemistry analyzer (DRI-CHEM NX500, FujiFilm Co., Saitama, Japan).

### Blood AA profiling

Determination of plasma AA concentrations was performed as described in detail previously (*43*). Briefly, plasma was deproteinized using sulfosalicylic acid and the supernatant was measured by high-performance liquid chromatography and fluorescence detection after precolumn derivatization with *o*-phthaldialdehyde.

### Histology and immunohistochemistry

Slices cut from paraffin-embedded liver sections (5 μm) were deparaffinized and rehydrated in xylene and ethanol, respectively. For collagen staining, tissue slides were incubated with picrosirius red (Sigma-Aldrich, UK) for 30 min after which picrosirius red was removed by vigorous shaking. Slides were then dehydrated and mounted with Vectamount permanent mounting medium (Vector Laboratories Ltd., UK).

TUNEL staining was performed using the In-Situ Cell Death Detection kit, POD (Roche, UK), as per the manufacturer’s protocol. Nuclei were counterstained with hematoxylin (Vector Laboratories Ltd., UK), after which sections were dehydrated and mounted with Vectamount permanent mounting medium (Vector Laboratories Ltd., UK). Image quantification was performed with ImageJ (version 1.53a) and expressed as the percentage of picrosirius red or TUNEL-positive area per total area.

### Multiplex immunofluorescence

Multiplex Immunofluorescence staining was performed as previously described (*44*). Formalin-fixed, paraffin-embedded liver tissue samples were deparaffinized and rehydrated in xylene (Roth, Germany) and ethanol (Roth, Germany). Antigen retrieval was performed with Tris-EDTA buffer (pH 9) in a water bath at 98°C for 30 min, followed by a cooling period of 20 to 30 min. Tissues were blocked with 2% normal goat serum (Thermo Fisher Scientific) to prevent nonspecific antibody binding. Slides were incubated overnight at 4°C with primary antibodies diluted in antibody dilution solution (Life Technologies, USA) and stained for 1 hour with fluorescently labeled secondary antibodies. After washing, the slides were incubated with 4′,6-diamidino-2-phenylindole (DAPI) (Sigma-Aldrich) nuclear counterstain for 5 min. After scanning the entire slide with a Zeiss Axio Observer7, images were merged, and the background was subtracted. After each run, antibodies were stripped by using the 2-mercaptoethanol/SDS method, and staining was repeated in multiple cycles over a 4-day period. Subsequently, all scans were aligned, hyperstacked, and concatenated using the plug in FIJI HyperStackReg V5.6. Fluorescence intensity measurement was performed using Image J.

### Liver protein extraction

Cryopreserved liver tissues were mechanically lysed (TissueLyser LT, Qiagen, UK) in mammalian protein extraction reagent (Thermo Fisher Scientific, UK) with a proteinase inhibitor cocktail (1X final concentration) (Sigma-Aldrich, UK). Lysates were centrifuged at 14,000 rpm for 10 min at 4°C, and supernatants were collected. Protein concentrations were determined using the Pierce BCA Protein Assay (Thermo Fisher Scientific, UK), according to the manufacturer’s protocol.

### Proteome profiler

The Proteome Profiler Mouse Apoptosis Array (R&D Systems, UK) was performed in liver tissues of the AA diet WT mouse model (groups: WT-NP, WT-AA, and WT-AA-OP), according to the manufacturer’s instructions. Protein lysates were pooled (100 μg per animal, *n* = 6 per group). The membranes were probed with 300 μg of protein per pool. Differential expression levels among the groups were semiquantitatively assessed with the Protein Array Analyzer macro for ImageJ.

### Western blot

Liver protein lysates for Western blot analysis were prepared as described above. Total protein lysates were randomly selected from each experimental group and pooled (100 μg per animal). The number of animals per pool is indicated in the figures and figure legends. Total protein concentration per pool was requantified using the Pierce BCA Protein Assay (Thermo Fisher Scientific, UK). For each pool, 20 μg of total liver protein was loaded onto a 4 to 12% Bis-Tris protein gel (Invitrogen, UK), separated by electrophoresis, and then transferred onto polyvinylidene difluoride membranes (Invitrogen, UK). Membranes were blocked in 5% nonfat dry milk powder (Sigma-Aldrich, UK) diluted in Tris-buffered saline containing 0.01% Tween 20 (TBS-T) for 1.5 hours. The blots were incubated overnight at 4°C with the primary antibody and, after being washed in TBS-T, incubated with a horseradish peroxidase–coupled anti-IgG (immunoglobulin G) antibody (Abcam, UK) diluted 1:10,000 at room temperature for 1 hour. Peroxidase activity was detected by SuperSignal West Pico chemiluminescent substrate (Thermo Fisher Scientific, UK). The FluorChem R System (ProteinSimple, CA, USA) was used for image acquisition. Antibody stripping was performed by incubating blots for 15 min with Restore Western blot Stripping Buffer (Thermo Fisher Scientific, UK) followed by washing in TBS-T. Densitometry was performed using ImageJ.

### OTC enzyme activity

OTC enzyme activity was determined by adding 10 μl of mouse liver protein sample at a concentration of 1 μg/μl to each well of a 96-well plate (in duplicate). The total volume of each well was adjusted to 100 μl with double distilled water. Blank wells and a standard curve of citrulline were set up. To each well, 25 μl of 50 mM ornithine, 2700 mM triethanolamine, and 150 mM carbamoyl phosphate was added. The plate was covered in aluminum foil and incubated at 37°C for 30 min. The enzymatic reaction was stopped by adding 80 μl of a phosphoric acid/sulfuric acid mixture (3:1) and 20 μl of 3% chromogenic reagent 2,3-butanedione monoxime. The plate was placed on a plate shaker at 37°C for 30 s and subsequently incubated at 95°C for 30 min. The rate of citrulline production was assessed spectrophotometrically at 490 nm.

### Liver metabolomics

Sample preparation was performed essentially as described previously (*45*) using ~25 mg of mouse liver tissue for metabolite extraction. After addition of 170 μl of ultrapure water, samples were homogenized and lysed using a sonication probe (5 × 10 s; Vibra Cell, Bioblock Scientific, Illkirich, France). Then, 20-μl aliquots of each sample were withdrawn for measuring their total protein concentration using a BCA Protein Assay kit (Thermo Fisher Scientific, Courtaboeuf, France). The remaining 150 μl of tissue lysate was mixed with 350 μl of methanol, and the resulting samples were centrifuged for 15 min at 4°C and 20,000*g* to remove the cell debris. After a further 90-min incubation on ice, a second centrifugation step was performed. Supernatants were recovered and split into two equal aliquots for C18 and HILIC analyses (see below). After evaporation to dryness under a stream of nitrogen using a TurboVap instrument (Thermo Fisher Scientific, Courtaboeuf, France), samples were stored at −80°C until analysis. Before liquid chromatography–mass spectrometry analysis, dried aliquots were resuspended in either 100 μl of water/acetonitrile (95:5, v/v) with 0.1% formic acid for C18 analysis or 100 μl of a mixture of 10 mM ammonium carbonate buffer (pH 10.5) and acetonitrile (40:60, v/v) for HILIC analysis (see below). Both reconstitution solvents contain a mixture of nine standards (i.e., ^13^C-glucose, ^15^N-aspartate, ethylmalonic acid, amiloride, prednisone, metformin, atropine sulfate, colchicine, and imipramine) to check for consistency of analytical results in terms of signal and retention time stability throughout the experiment. Quality control samples were constituted by mixing 20-μl aliquots of each sample and were injected every 10 samples throughout the acquisition series for further data normalization/standardization purposes.

Untargeted metabolomics experiments were performed by LC-HRMS, using a combination of two complementary chromatographic methods (*46*, *47*) consisting of reversed-phase chromatography (C18 chromatographic column) and HILIC for the analysis of hydrophobic and polar metabolites, respectively. LC-HRMS experiments were performed by using an Ultimate 3000 chromatographic system (Thermo Fisher Scientific, Courtaboeuf, France) coupled to a Q Exactive mass spectrometer from Thermo Fisher Scientific fitted with an electrospray source and operating in the positive and negative ion modes for metabolite separations on C18 and HILIC columns (see below), respectively. Chromatographic conditions were exactly those previously described by our group (*46*), whereas the mass spectrometer was operated with capillary voltage at −3 kV in the negative ionization mode and 3 kV in the positive ionization mode and a capillary temperature set at 280°C. The sheath gas pressure and the auxiliary gas pressure were set at 60 and 20 arbitrary units with nitrogen gas, respectively. The detection was achieved from *m/z* (mass/charge ratio) 70 to 1000 at 140,000 resolution in both ionization modes.

Data processing and some statistical analyses were performed using the Workflow4Metabolomics (W4M) platform (*48*). Metabolite features were first annotated according to accurate measured masses and chromatographic retention times by using our spectral database (*47*). Metabolite identification was further confirmed using tandem mass spectrometry (MS/MS) data collected under nonresonant collision-induced dissociation conditions using higher-energy C-trap dissociation. MS/MS data were further matched both manually and automatically using the MS-DIAL software to the spectra included in our in-house spectral database, as described previously (*45*, *49*). To be identified, metabolites had to match at least two orthogonal criteria (i.e., accurate measured mass, retention time, and MS/MS spectrum) to those of an authentic chemical standard analyzed under the same analytical conditions, as proposed by the Metabolomics Standards Initiative (*50*).

### Liver mRNA expression of UCEs

Total RNA was extracted from snap-frozen liver tissue and cleaned by using the QIAzol lysis protocol (Qiagen, UK). Quantification of total RNA was performed using the NanoDrop1000 System (Thermo Fisher Scientific, USA) and was followed by gel electrophoresis for evaluation of RNA integrity. Subsequently, for each sample, 0.25 μg of total RNA was retrotranscribed using the QuantiTect Reverse Transcription Kit (Qiagen, CA, USA). Then, 1 μl of cDNA sample was used for the real-time polymerase chain reaction (PCR) using the ABI 7500 Fast Real-Time PCR System (Thermo Fisher Scientific, UK). Individual TaqMan assays for the target genes (i.e., CPS1 and OTC) were used as appropriate, and B2M was used as the housekeeping gene. Each sample was tested in duplicate. Relative quantification of the target genes was carried out using the 2^−ΔΔCt^ method in which the sham rats served as the reference group. Target genes were normalized for B2M expression.

### In vitro studies with immortalized human hepatocytes (HHL5)

HHL5 cells were used to assess whether ammonia directly induces hepatocyte injury and cell death. The 96-well plates were seeded with 200 μl of a cell suspension (1.3 × 10^6^ cells/ml) and cultured for 24 hours. Cell culture medium was then exchanged with 50 μl of culture medium containing 50, 100, or 300 μM NH_4_Cl and incubated at 37°C for another 24 hours.

Cell viability was assessed using the CellTiter-Glo Luminescent Cell Viability Assay (Promega, USA), according to the manufacturer’s instructions. Briefly, 100 μl of the CellTiter-Glo Reagent was added to each well. The well plate was then incubated for 2 min on an orbital shaker and subsequently incubated for 10 min at room temperature. Luminescence was recorded using an integration time of 0.5 s per well.

To assess the presence of cell death, the Cell Death Detection ELISA^PLUS^ (Roche, UK) was used. First, cell culture supernatants were collected and centrifuged for 10 min at 200*g*, after which the supernatant was collected and kept on ice. The cells were then lysed using 100 μl of the lysis buffer included in the kit and incubated for 30 min at room temperature. The cells were then centrifuged for 10 min at 200*g*, and the supernatant was collected and kept on ice. The ELISA was performed using both the cell lysates and supernatants according to the manufacturer’s protocol. The amount of cell death was determined colorimetrically at 420 to 480 nm by spectrophotometry.

### In vitro studies with HEK-Blue hTLR4 reporter cells

To assess whether ammonia can directly activate TLR4, the HEK-Blue hTLR4 reporter cells (InvivoGen, California, USA) were used according to the manufacturer’s protocol. Briefly, 200 μl of a cell suspension (125,000 cells/ml) was seeded in a 96-well plate (20,000 cells per well) and incubated for 48 hours at 37°C. Cells were then incubated with different concentrations of NH_4_Cl (0, 1.25, 2.5, 5, 10, 25, 50, 100, 500, and 1000 μM) for 24 hours. Secreted alkaline phosphatase (SEAP) was detected by the addition of 20 μl of supernatant to 180 μl of alkaline phosphatase detection medium (QUANTI-Blue, InvivoGen, USA). After 4 hours of incubation at 37°C, SEAP activity was determined colorimetrically at 650 nm by spectrophotometry. Samples and controls were run in triplicate.

### In vitro studies with PMHs

The method for isolating PMHs followed a well-established protocol with multiple steps. Initially, the liver of C57B6/J mice (aged 18 to 23 weeks) underwent Ca^2+^-free perfusion to eliminate blood and nonparenchymal cells from the liver tissue, after which collagenase perfusion was performed. The hepatocytes were then refined to achieve over 90% viability using a Percoll gradient (*51*). The purified hepatocytes were subsequently seeded at a density of 10,000 cell per well onto cell culture plates precoated with collagen type 1 (5 μg/cm^2^). For sustenance, the hepatocytes were cultured in William’s E-medium, which was supplemented with 5% fetal bovine serum, 2 mM l-glutamine, 5% Penstrep (penicillin-streptomycin), and hepatocyte growth supplements (CM4000) sourced from Thermo Fisher Scientific. This enriched medium provided all the necessary nutrients, growth factors, and antibiotics to ensure the effective growth and maintenance of the PMHs in vitro.

The assessment of OCRs was measured in PMHs as described previously (*52*). Briefly, PMHs were cultured overnight, and the next day, these cells were treated with a preoptimized dosage of ammonium chloride (5 mM), with or without TAK-242 (0.2 μM) for 24 hours and incubated in a humidified incubator set to 37°C with 5% CO_2_. For the OCR, the culture medium was switched to XF basal medium from Agilent Technologies, enhanced with 10 mM glucose, 1 mM pyruvate, and 1 mM glutamine. The cells were then incubated at 37°C in a nonhumidified incubator without CO_2_ for 1 hour. To evaluate the OCR, specific inhibitors [oligomycin at 2 μM, FCCP (carbonyl cyanide *p*-trifluoromethoxyphenylhydrazone) at 1 μM, rotenone at 1 μM, and antimycin at 1 μM] were titrated and injected through designated ports A, B, and C of the Bioanalyzer. The resulting data were normalized to consider the total number of cells in each well. For the fuel dependency assay, a preoptimized dosage of 5 μM mitochondrial pyruvate carrier inhibitor (UK-5099), carnitine palmitoyltransferase-1 inhibitor (etomoxir), and glutaminase inhibitor (BPTES) was used.

### Statistics

Data analysis and graph preparation was performed using the PRISM software (GraphPad, USA, version 9). In case of a Gaussian distribution, data with multiple groups were compared using one-way ANOVA with Tukey post hoc test. In case of a skewed distribution, a Kruskal-Wallis test with post hoc Dunn’s test was performed. Data are shown as means ± SD or median with interquartile range, as indicated in the figure legends. A *P* value of <0.05 was considered statistically significant. In the figures, significance is shown as *P < 0.05, **P < 0.01, ***P < 0.001, and ****P < 0.0001. For the sample size calculation, we anticipated a 50% decrease in circulating ammonia in the treatment groups with an alpha error of 0.05 and power of 80%, resulting in a minimum sample size of five animals per group. Of all animals included in the present experiments (*n* = 150), 11 (7.3%) were excluded because of significant hemolysis of the plasma sample, which is known to interfere with ammonia measurements (*53*, *54*). After centrifugation, individual plasma samples were visually examined for hemolysis by comparing the appearance of the serum with a specimen integrity chart based on color. In case of moderate or gross hemolysis (>200 mg/dl), the sample was excluded from the biochemistry analyses. For any of the other experiments, no outliers were excluded. For the analysis of the liver metabolomics, PCA and heatmaps were performed and plotted with PRISM and the free online web-based platform MetaboAnalyst 5.0 (*55*).

## Supplementary Materials

### The PDF file includes:

Supplementary Results Figs. S1 to S8 Table S1 Legends for tables S2 to S6 Full Western blot gels References
