# Supplementary material for: Hyperammonemia induces programmed liver cell death
Source: Sci Adv. 2025 Mar 7;11(10):eado1648. doi: 10.1126/sciadv.ado1648 (PMC11887801; doi:10.1126/sciadv.ado1648)
Supplement: Supplementary file 1 — Supplementary Results Figs. S1 to S8 Table S1 Legends for tables S2 to S6 Full Western blot gels References [file sciadv.ado1648_sm.pdf]

Science Advances
AAAS

Supplementary Materials for

Hyperammonemia induces programmed liver cell death

Annarein J. C. Kerbert *et al.*

Corresponding author: Annarein J. C. Kerbert, j.c.kerbert@lumc.nl; Rajiv Jalan, r.jalan@ucl.ac.uk

Sci. Adv. **10**, eado1648 (2025)
DOI: 10.1126/sciadv.ado1648

The PDF file includes:

Supplementary Results
Figs. S1 to S8
Table S1
Legends for tables S2 to S6
Full Western blot gels
References

Other Supplementary Material for this manuscript includes the following:

Tables S2 to S6

Supplementary results

Blood amino acid profiling

Plasma amino acid profiling was performed to validate the intake of the AA diet by the mice and to investigate the effects on plasma concentrations of amino acids involved in ammonia metabolism. Nearly all amino acids that were included in the AA diet (please see Table S1 for the AA diet composition), were increased in the plasma of WT-AA and TLRKO-AA mice (Fig. S3a). For some of these amino acids, there was a trend for decreased plasma concentrations in the OP and TAK-242 treated mice (i.e., histidine, valine, phenylalanine, tryptophan, alanine and leucine).

In contrast, plasma concentrations of amino acids that were *not* included in the AA diet were generally reduced in the WT-AA and TLR4KO-AA group as compared to WT-NP and TLR4KO-NP, respectively (Fig. S3b). Significantly reduced concentrations were observed for plasma isoleucine and methionine in TLR4KO-NP vs. TLR4KO-AA ($p=0.013$ and $p<0.0001$, respectively).

We separately analysed the plasma concentrations of amino acids that are involved in the urea cycle and ammonia metabolism (Fig. S3c). Except for arginine, these amino acids were not included in the AA diet. We especially observed a significant increase in plasma ornithine in the WT-AA mice as compared to WT-NP (304.8 ± 103.3 mmol/L vs. 121.0 ± 25.67, $p=0.0026$). Plasma ornithine levels were significantly reduced in both OP and TAK-242 treated mice as compared to WT-AA (WT-AA-OP: 156.9 ± 74.78 mmol/L, $p=0.0168$; WT-AA-TAK-242: 170.5 ± 37.71 mmol/L, $p=0.0305$). Interestingly, no significant increase in plasma ornithine was observed between TLR4KO-NP and TLR4KO-AA mice. TLR4KO-AA mice also had significantly lower plasma ornithine concentrations as compared to WT-AA (132.7 ± 1002 mmol/L vs. 304.8 ± 103.3 mmol/L, $p=0.0053$). Ornithine stimulates the enzyme carbamoyl-phosphate synthetase I (CPS1), which catalyzes the rate-limiting step of the urea cycle (CO_2 + NH_4+ → Carbamoyl phosphate). Therefore, ornithine availability is often described as limiting the cycle flux and is a key metabolite in ammonia detoxification. Its accumulation in hyperammonemia may point towards urea cycle dysfunction. No significant changes among groups were found for the two other assessed urea cycle intermediates, plasma citrulline and arginine.

As for ornithine, we observed a similar pattern for plasma concentrations of glutamine and glutamate, although there were no significant changes among groups. There was, however, a clear trend of increased plasma levels of both amino acids in WT-AA and TLR4KO-AA as compared to their control groups (Fig. S3c). TAK-242 treatment led to a reduction of both glutamine and glutamate as compared to WT-AA, which was most pronounced for glutamate ($p=0.0732$).

Liver metabolomics: metabolites related to polyamine synthesis.

Among the differential metabolites in the liver metabolomics analysis (WT-NP vs WT-AA) were metabolites involved in polyamine and pyrimidine synthesis pathways, which are both interrelated with the urea cycle.

Polyamine synthesis begins with ornithine, which is decarboxylated by ornithine decarboxylase (ODC) to produce the first polyamine, putrescine. Subsequently, spermidine and spermine are synthesized by the sequential addition of aminopropyl groups to putrescine. We observed a significant increase in hepatic putrescine in WT-AA as compared to WT-NP ($p=0.012$), which was prevented by OP and TAK-242 treatment ($p=0.0003$ and $p=0.002$ as compared to WT-AA, respectively; Fig. S4). A very similar pattern among the treatment groups was observed for acetylated spermidine (N-acetyl-spermidine). The significant increase of N-acetyl-spermidine in WT-AA may be due to increased activity of N1-acetyltransferase (SAT1), an enzyme which is involved in recycling of polyamines and can undergo superinduction in the presence of reactive oxygen species (ROS) (*56*). Acetylation is the rate-limiting step in polyamine catabolism and maintains intracellular levels through transportation out of the cell or recycling by oxidation to a

lower polyamine. Excess of N-acetyl-spermidine may therefore further contribute to the elevated putrescine levels. Similar levels of spermidine were observed in WT-AA as compared to WT-NP (Fig. S4). However, OP and TAK-242 treatment led to a significant reduction in spermidine levels as compared to WT-AA ($p<0.0001$ and $p=0.0003$, respectively). A similar pattern was observed for spermine, although changes were not significant.

In the TLR4KO mice, no significant changes in metabolites related to polyamine metabolism were observed.

Liver metabolomics: metabolites related to pyrimidine and purine synthesis.

Hyperammonemia in WT-AA was found to be associated with increased hepatic concentrations of key intermediates of the pyrimidine synthesis pathway. The pyrimidine biosynthesis pathway is initiated by the formation of carbamoyl phosphate. In hepatocytes, there are two distinct pools of carbamoyl phosphate, one in the mitochondria (participating in the urea cycle) and one in the cytosol (participating in the pyrimidine synthesis pathway). They require the action of two different isozymes of CPS: CPS1 and CPS2. CPS1 is mainly present in hepatocytes and participates in the urea cycle, whereas CPS2 is present in all cells and participates in the pyrimidine synthesis pathway. The cytosolic form of carbamoyl phosphate is primarily formed by ammonia derived from glutamine. However, in the setting of insufficient capacity for ammonia detoxification, mitochondrial carbamoyl phosphate leaves the mitochondria and enters the pyrimidine pathway. The first three reactions are carried out by the multifunctional CAD protein, which contains active sites for CPS2, aspartate transcarbamylase (ATC) and dihydroorotase to form 1) carbamoyl phosphate + glutamate aspartate, 2) carbamoylaspartic acid and 3) dihydroorotic acid, respectively. The fourth enzyme, dihydroorotate dehydrogenase (DHODH), catalyzes the oxidation of dihydroorotic acid to orotic acid. Orotic acid is then converted to orotidine monophosphate (OMP) and uridine monophosphate (UMP) by orotate phosphoribosyltransferase and orotidine 5-phosphate decarboxylase, respectively. Finally, UMP is dephosphorylated to uridine by nucleotidase or formed from cytidine by cytidine deaminase. In WT-AA, we observed significantly increased levels of both dihydroorotic acid ($p=0.039$) and orotic acid ($p<0.0001$) as compared to WT-NP (Fig. S4). TAK-242 and OP treatment significantly reduced hepatic orotic acid levels as compared to WT-AA (both $p<0.0001$), whereas this was not the case for dihydroorotic acid. This seemingly increased flux through the pyrimidine synthesis pathway in WT-AA as compared to WT-NP resulted in significantly increased levels of the pyrimidine cytidine ($p=0.0375$), whereas uridine concentrations were significantly decreased in WT-AA as compared to WT-NP ($p<0.0001$). OP and TAK-242 treatment led to an even further decrease in uridine levels as compared to WT-AA ($p=0.0002$ and $p=0.00028$, respectively).

For the purine synthesis pathway, no significant changes in metabolites were observed between WT-NP and WT-AA (Fig. S4). There was a trend towards a decrease in inosine production in WT-AA as compared to WT-NP, which was restored with OP and TAK-242 treatment ($p=0.005$ and $p<0.0001$, respectively).

In the TLR4KO mice, no significant changes in metabolites related to pyrimidine and purine metabolism were observed between TLRKO-NP and TLR4KO-AA (Fig. S4).

Liver metabolomics: metabolites related to mitochondrial beta-oxidation and Krebs cycle.

Changes in metabolites involved in mitochondrial beta-oxidation were observed. The carnitine shuttle is essential for the transport of long chain fatty acids into the mitochondria where they undergo oxidation. Defects in fatty acid oxidation or the carnitine cycle can lead to hyperammonemia by inhibition of the urea cycle via multiple pathways (*57-59*). Increased levels of acetyl-carnitines are considered markers of metabolic dysfunction and can be a consequence of reduced mitochondrial activity (*60-62*). In the current study, carnitine, acetyl-carnitine and propionyl-carnitine were all significantly increased in WT-AA compared to WT-NP ($p=0.0002$,

p=0.008 and p=0.0054, respectively), which was prevented by OP and TAK-242 treatment (Fig. S4).

Among the metabolites involved in the Krebs cycle, a significant increase in malic acid was observed in WT-AA as compared to WT-NP ($p<0.001$), which was prevented by OP and TAK-242 treatment (Fig. S4), which may reflect disturbances in mitochondrial metabolism (*63*, *64*). We observed no changes in pyruvic acid and citric acid among groups.

No changes in metabolites related to mitochondrial beta-oxidation and the Krebs cycle were observed in TLR4KO-AA vs. TLR4KO-NP (Fig. S4).

Supplementary figures

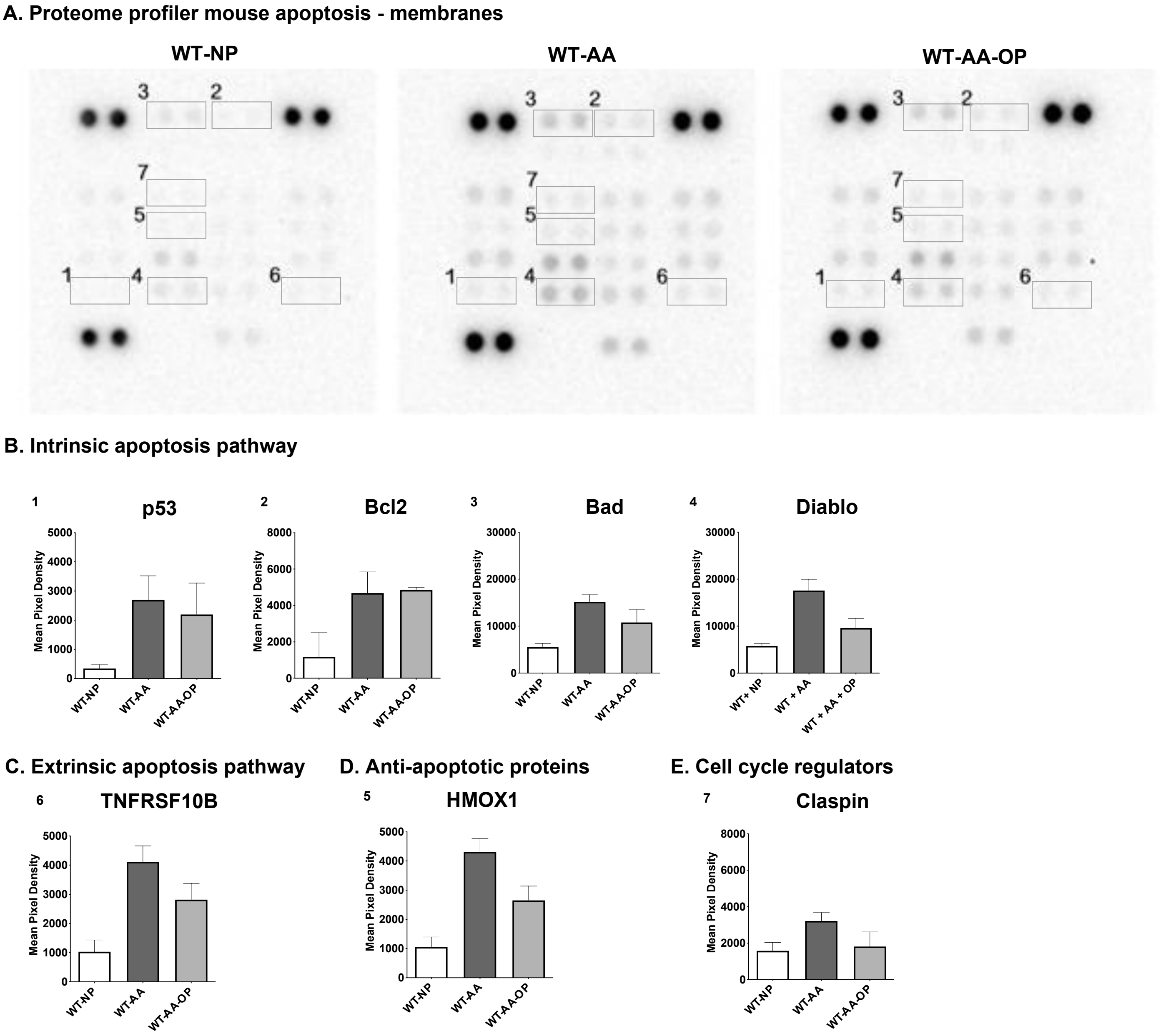

Fig. S1. Proteome profiler mouse apoptosis array. (A) The numbered and outlined spots on the membranes resemble the apoptotic proteins that were found to be regulated in WT-AA as compared to WT-NP. **(B)** Especially expression of proteins involved in the intrinsic apoptosis pathway were found to be affected by hyperammonemia. In addition, hepatic expression of proteins involved in the extrinsic apoptosis pathway **(C)**, the anti-apoptotic protein HMOX1 **(D)** and the cell-cycle regulator claspin **(E)**, were found to be increased in the setting of hyperammonemia.

Data are presented as mean ± standard deviation. Bars represent pooled samples (n=6 per group).

Abbreviations: AA, amino acid diet; NP, normal powdered diet; OP, ornithine transcarbamylase; WT, wild type.

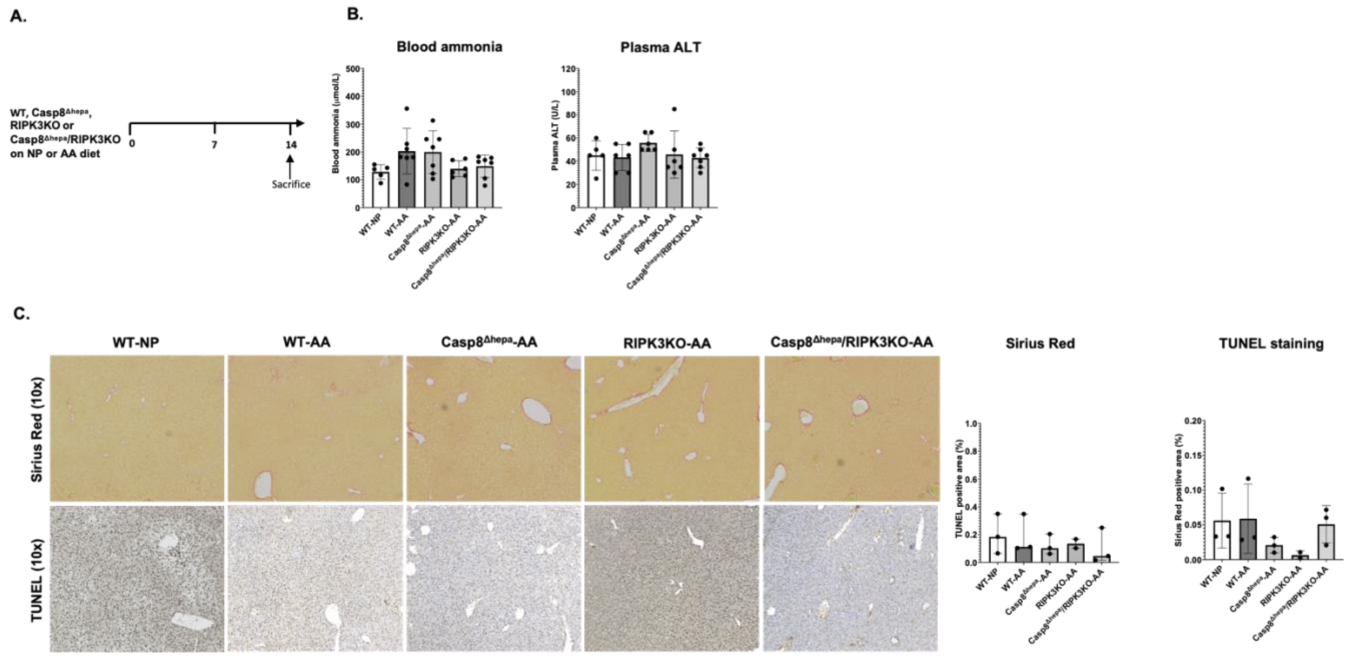

Fig. S2. Hyperammonemia in Casp8$^{\Delta hepa}$, RIPK3KO and Casp8$^{\Delta hepa}$/RIPK3KO mice. (**A**) Study design. The AA-diet was applied in Casp8$^{\Delta hepa}$, RIPK3KO and Casp8$^{\Delta hepa}$/RIPK3KO to validate our findings that treatment with a RIPK1 inhibitor (RIPA-56) prevents against hyperammonemia and liver injury in WT-AA mice. In addition, we aimed to identify whether caspase-8 dependent apoptosis or RIPK3-dependent necroptosis is the primary mode of cell death in hyperammonemia. (**B**) Bar graphs showing circulating ammonia and ALT levels. We did not observe significant changes in blood ammonia and plasma ALT among the experimental groups, although there was a trend towards a reduction in ammonia levels in RIPK3KO mice as opposed to Casp8$^{\Delta hepa}$ mice. (**C**) Microscopy and quantification of Sirius Red and TUNEL staining. No fibrogenesis and cell death was observed in the different experimental groups. *Groups are compared by ordinary one-way ANOVA with post-hoc Tukey HSD test or Kruskal Wallis test with post-hoc Dunn's test. ns, non-significant, *p<0.05, ****p<0.0001.*

Abbreviations: ALT, alanine aminotransferase; AA, amino acid diet; KO, knock-out; NP, normal powdered diet; RIPK3, receptor-interacting protein kinase 3; WT, wild type.

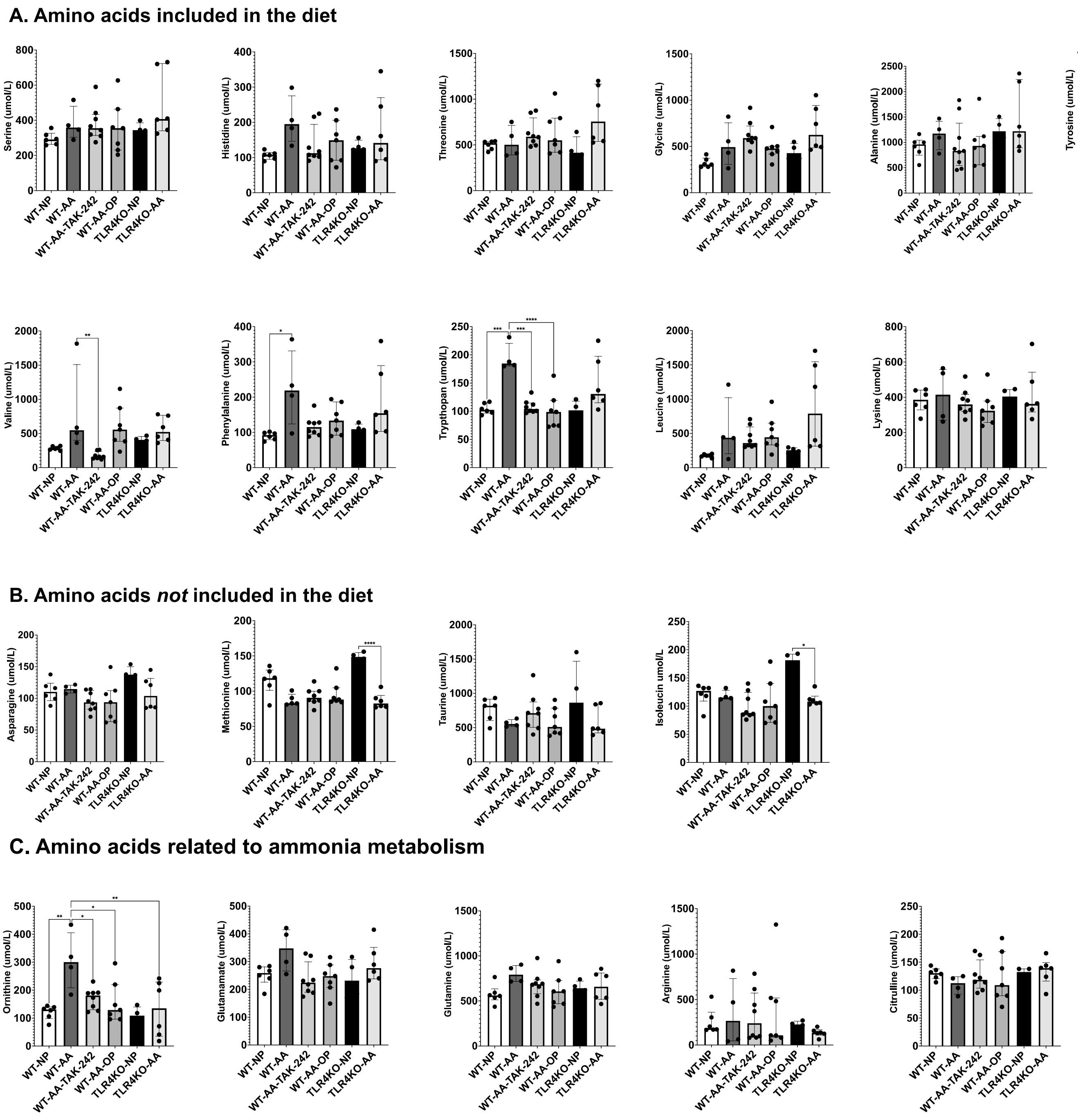

Fig. S3: Plasma amino acid profiling. (A) Plasma amino acid profiling was performed to validate the intake of the AA diet by the mice and to investigate the effects on plasma concentrations of amino acids involved in ammonia metabolism. Nearly all amino acids that were included in the AA diet, were increased in the plasma of WT-AA and TLRKO-AA mice. **(B)** In contrast, plasma concentrations of amino acids that were *not* included in the AA diet were generally reduced in the WT-AA and TLR4KO-AA group. Significantly reduced concentrations were observed for plasma isoleucine and methionine in TLR4KO-NP vs. TLR4KO-AA. **(C)** Among the amino acids that are related to ammonia metabolism, especially a profound increase in plasma ornithine was observed in WT-AA, which was significantly reduced by OP treatment or TLR4 inhibition. *All data are presented as mean ± standard deviation. Groups are compared by ordinary one-way ANOVA with post-hoc Tukey HSD test. ns, non-significant, *p<0.05, **p<0.01, ***p<0.001, ****p<0.0001. Abbreviations: AA, amino acid diet; NP, normal powdered diet; OP, ornithine phenylacetate; WT, wild type.*

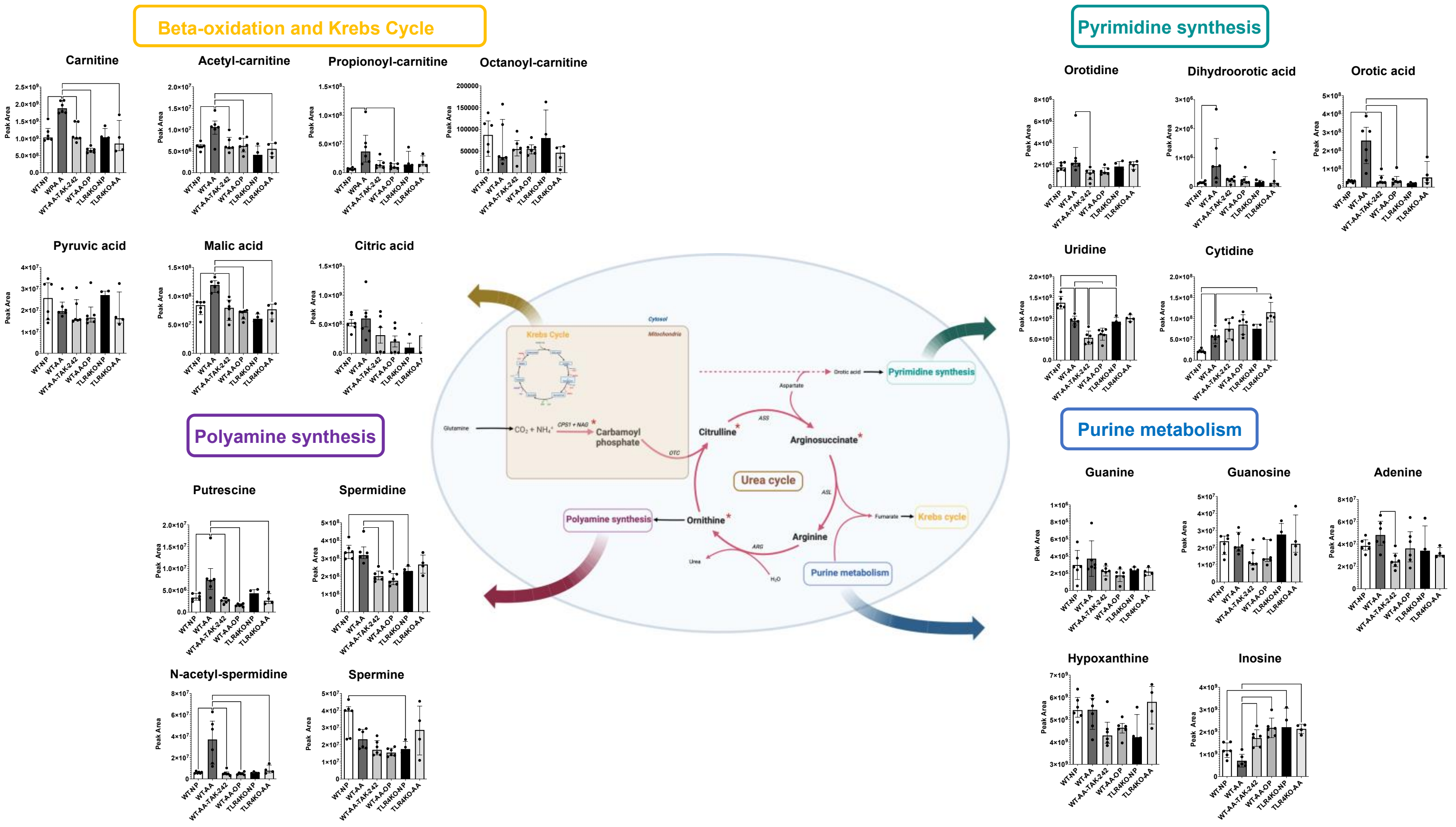

Fig. S4. Impact of hyperammonemia on metabolic pathways interrelated with the urea cycle. Bar graphs representing peak areas of individual metabolites that were among the top-50 metabolites in the metabolomics analysis. Metabolites are involved in several metabolic pathways that are interrelated with the urea cycle. The urea cycle provides precursors for both polyamine and pyrimidine synthesis. We observed a significant increase in metabolites associated with polyamine (purple) and pyrimidine synthesis (blue) during hyperammonemia, which was prevented by OP and TAK-242 treatment. Hyperammonemia also induced changes in other mitochondrial metabolic pathways. Dysfunction of beta-oxidation (yellow) was indicated by increased levels of metabolites involved in the carnitine shuttle. Among metabolites involved in the Krebs cycle (yellow), hyperammonemia was associated with increased levels of malic acid, which is likely to be a consequence of increased hepatic argininosuccinic acid, which is broken down into arginine and fumaric acid in the urea cycle.

*Data are presented as mean ± standard deviation. Groups are compared by ordinary one-way ANOVA with post-hoc Tukey HSD test. ns, non-significant, *p<0.05, **p<0.01, ***p<0.001, ****p<0.0001.*

Abbreviations: AA, amino acid diet; NP, normal powdered diet; OP, ornithine phenylacetate; WT, wild type.

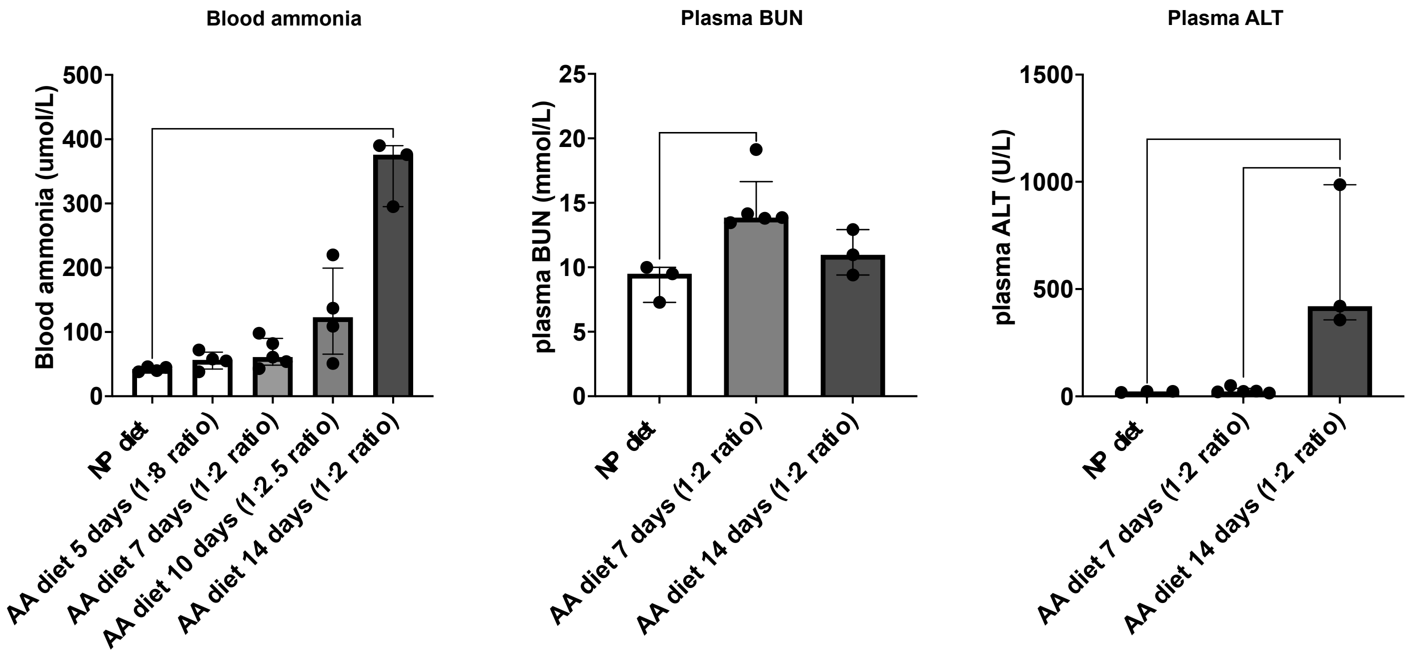

Fig. S5. Dose finding studies of the AA diet to induce chronic hyperammonemia in wild type C57BL/6 mice. An AA:NP diet ratio of 1:2 for the duration of 14 days was defined as the optimal formula for this model.

*Data are presented as mean ± standard deviation. Groups are compared by ordinary one-way ANOVA with post-hoc Tukey HSD test. ns, non-significant, *p<0.05, ****p<0.0001.*

Abbreviations: AA, amino acid; BUN, blood urea nitrogen; NP, normal powdered.

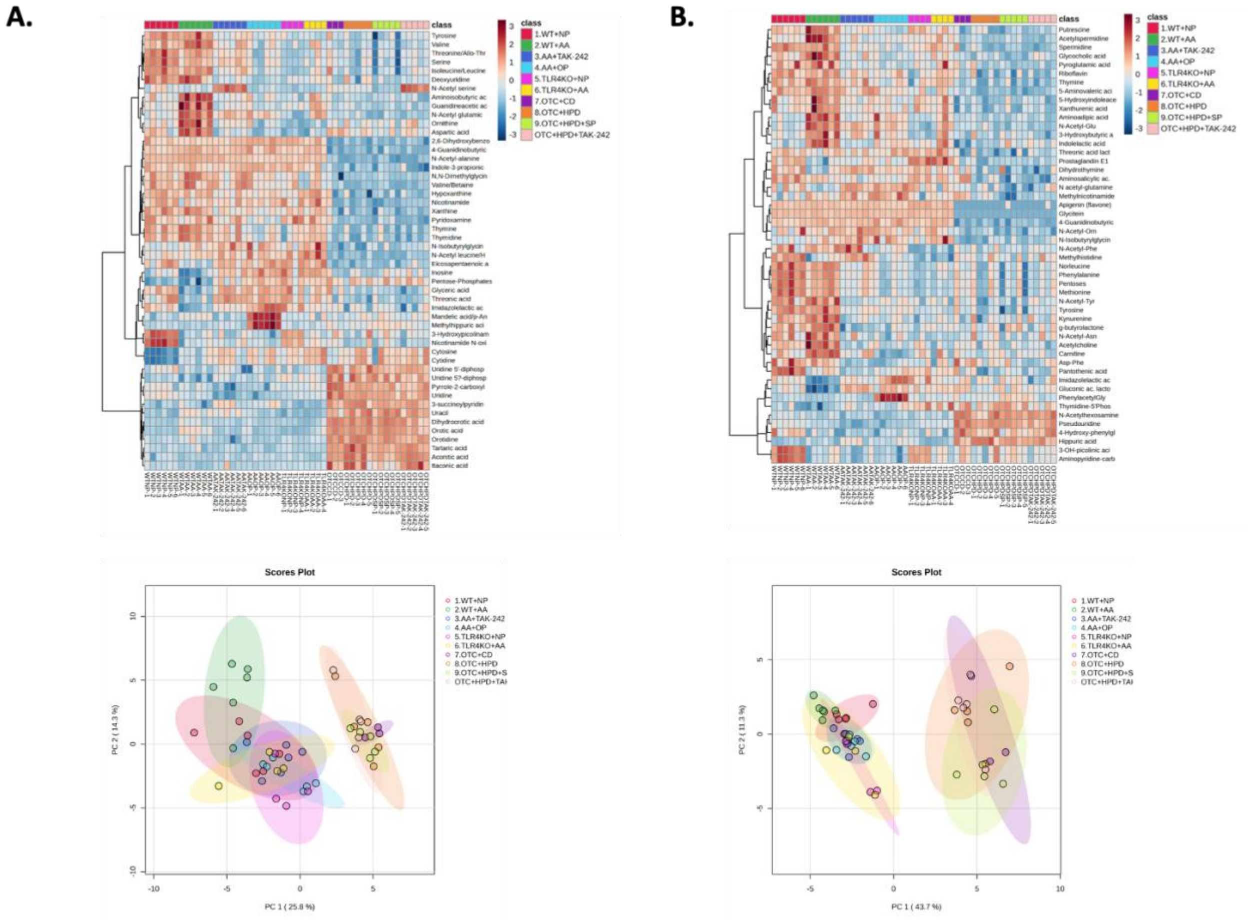

Fig. S6. Impact of hyperammonemia on liver metabolomics in OTC$^{spf\text{-}ash}$ mice. This figure is showing heatmaps including the top-50 differential metabolites in the liver metabolomics analysis, according to ANOVA p-values <0.05 between WT-AA and WT-NP. All analyzed groups (i.e., WT, TLR4KO and OTC$^{spf\text{-}ash}$ mice) are shown. Data of the WT and TLR4KO mice are further analyzed and described in the main manuscript and Fig. 3 and S4. Heatmaps and PCA plots for (**A**) HILIC data and (**B**) C18 data show that the OTC$^{spf\text{-}ash}$ mice have a distinct metabolic profile from WT and TLR4KO mice, but there were no differences among the groups in this model.

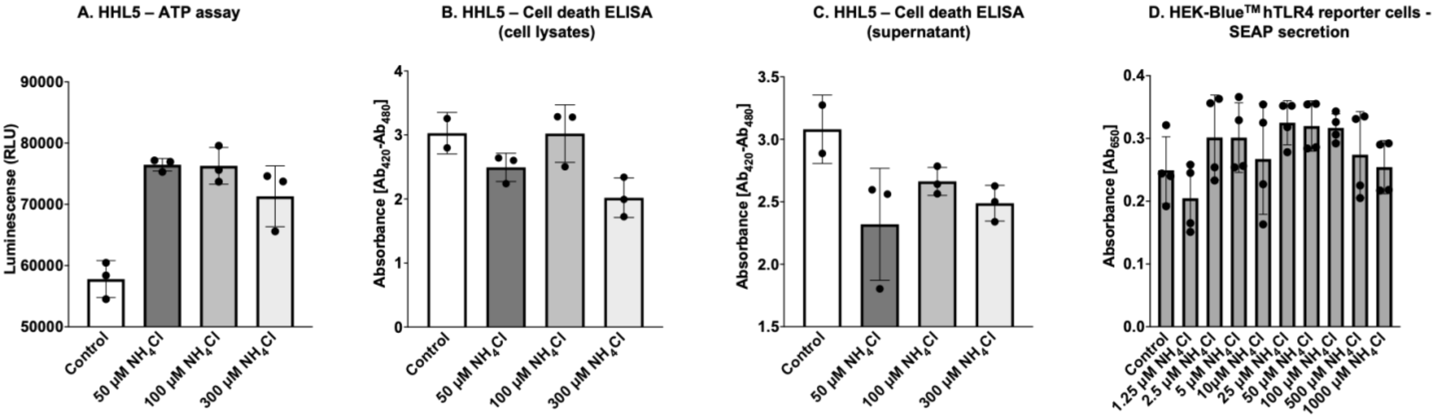

Fig. S7. Impact of ammonium chloride on hepatocyte cell viability and cell death and on TLR4 transactivation. (**A**) Bar graphs representing results of an ATP assay in HHL5 hepatocytes exposed to different concentrations of NH_4Cl. Clinically relevant concentrations of NH_4Cl were found not to impact on cell viability. (**B, C**) Bar graphs representing the results of a cell death ELISA in cells and supernatant of HHL5 cells exposed to NH_4Cl. No hepatocyte cell death was observed following incubation with clinically relevant concentrations of NH_4Cl. (**D**) Bar graphs showing assessment of SEAP secretion after incubation of HEK-TLR4 reporter cells with a wide range of NH_4Cl concentrations. No TLR4 transactivation upon incubation with NH_4Cl was observed.

Data are presented as median with interquartile range. Groups are compared by Kruskal Wallis test and post-hoc Dunn's test.

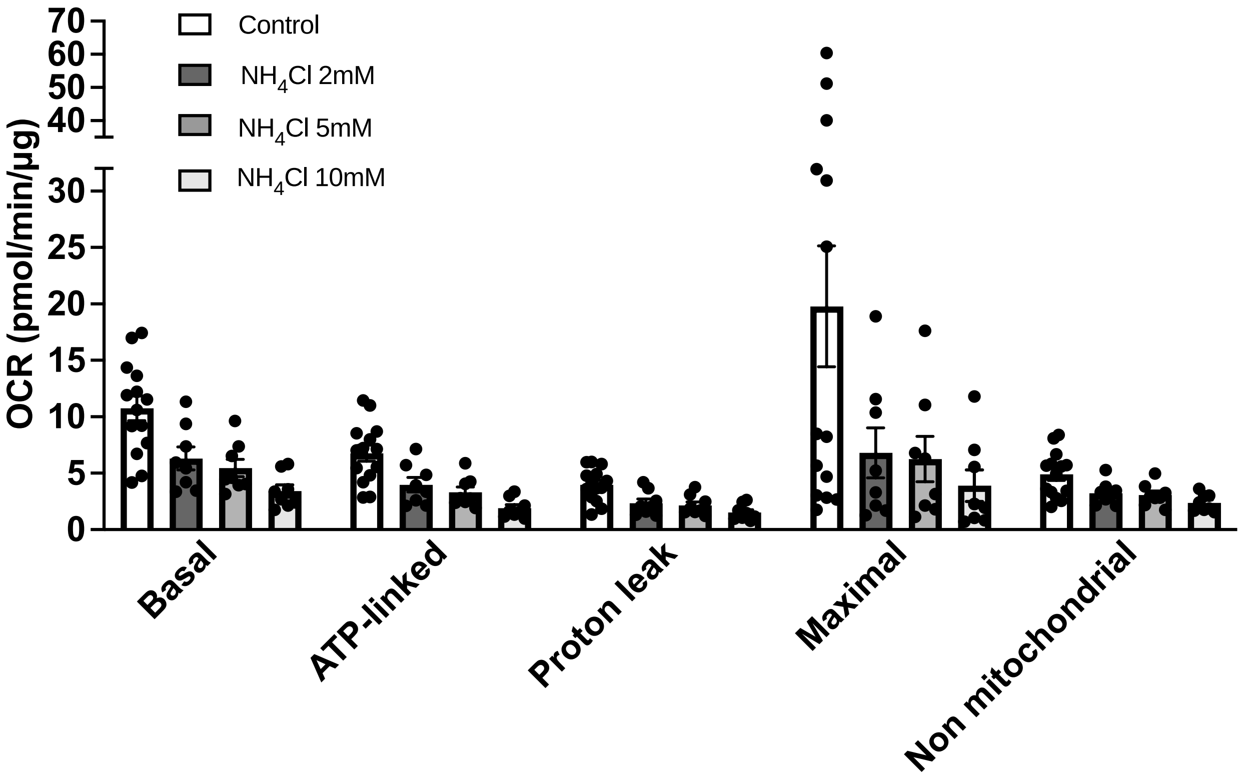

Fig. S8. Ammonium chloride induces a dose-dependent decrease in OCR. Bar charts showing mitochondrial respiration changes measured by Seahorse flux analyzer in PMHs exposed to 2, 5 and 10mM NH_4Cl. OCR was analyzed for basal respiration, ATP-linked respiration, proton leak respiration, maximal respiration, and non-mitochondrial oxygen consumption. The results show a non-significant, dose-dependent decrease in OCR of PMHs upon exposure to the different concentrations of NH_4Cl. *Data are presented as mean ± standard deviations. Groups are compared by ordinary one-way ANOVA with post-hoc Tukey test.*

Supplementary tables

**Amino acid (AA)**	**Concentration in AA mixture (%)**	**Concentration in NP diet (%)**	**Final concentration in AA diet (AA:NP mixture= 1:2) (%)**
Leucine	15	0,98	5,65
Phenylalanine	7,7	0,66	3,01
Glutamate	7	3,17	4,45
Alanine	10	0,16	3,44
Proline	4,4	1,2	2,27
Threonine	5,8	0,49	2,26
Aspartate	11	0,67	4,11
Serine	5	0,56	2,04
Glycine	4,8	1,11	2,34
Arginine	3,3	0,91	1,71
Lysine	9,6	0,66	3,64
Histidine	8,4	0,35	3,03
Tyrosine	3	0,49	1,33
Tryptophan	1,5	0,18	0,62
Valine	10,6	0,69	3,99
Methionine	0	0,22	0,15
Cysteine	0	0,24	0,16
Isoleucine	0	0,54	0,36

Table S1. Diet composition. Concentrations (%) of amino acids in the AA mixture, NP diet and AA diet. The AA diet was prepared by adding the AA mixture to the NP diet in a 1:2 ratio. *Abbreviations: AA, amino acid; NP, normal powdered.*

Supplemental auxiliary files

Table S2. Full list of annotated metabolites.

Table S3. HILIC dataset – WT and TLR4KO mouse model.

Table S4. C18 dataset – WT and TLR4KO mouse model.

Table S5. HILIC dataset – WT, TLR4KO and OTC$^{spf\text{-}ash}$ mouse model.

Table S6. C18 dataset – WT, TLR4KO and OTC$^{spf\text{-}ash}$ mouse model.

Full Western Blot membranes

Blots from Fig. 3a.:

Bax, 21 kDa

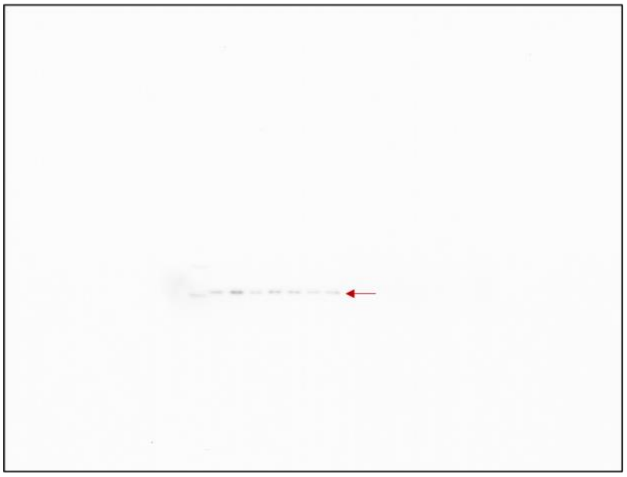

Bcl2, 26 kDa

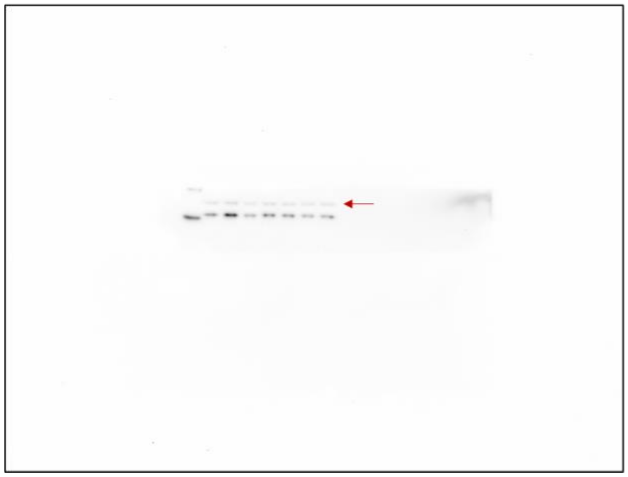

Cleaved Caspase-3, 18 kDa

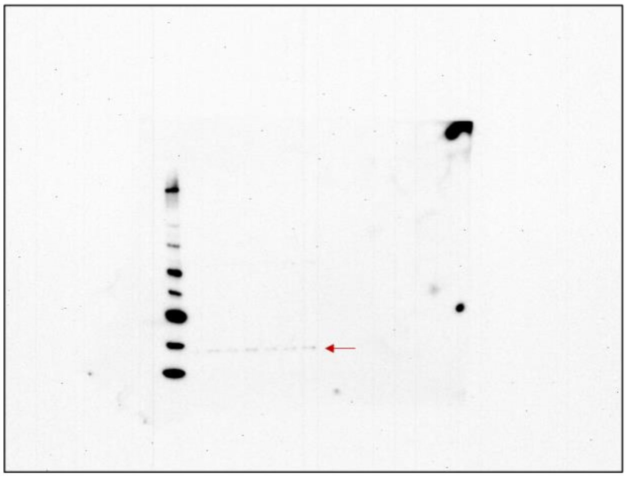

Diablo, 21 kDa

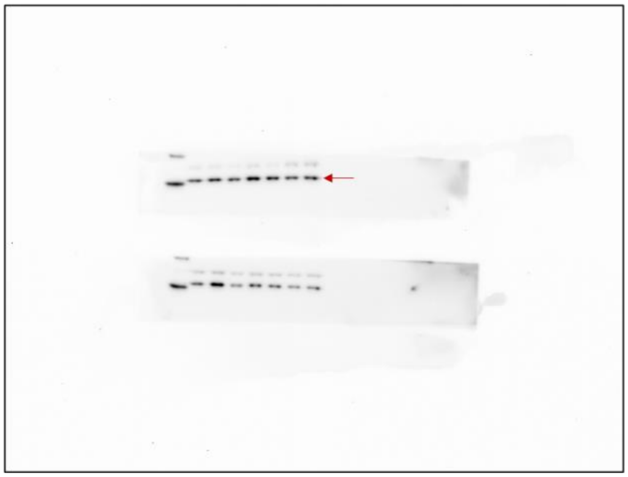

GAPDH 2, 37 kDa

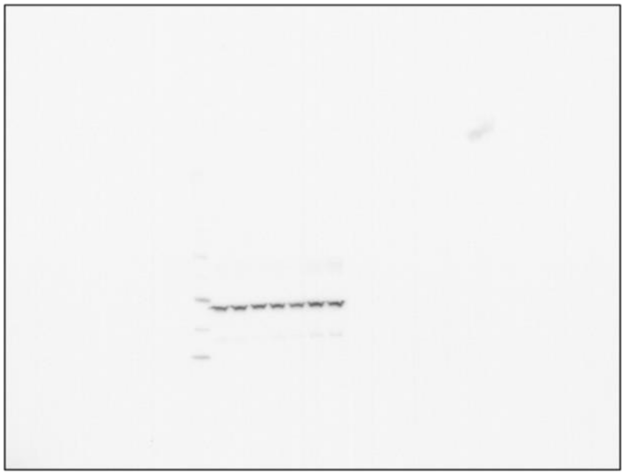

Membranes from Fig. 3b:

RIPK1, 76 kDA

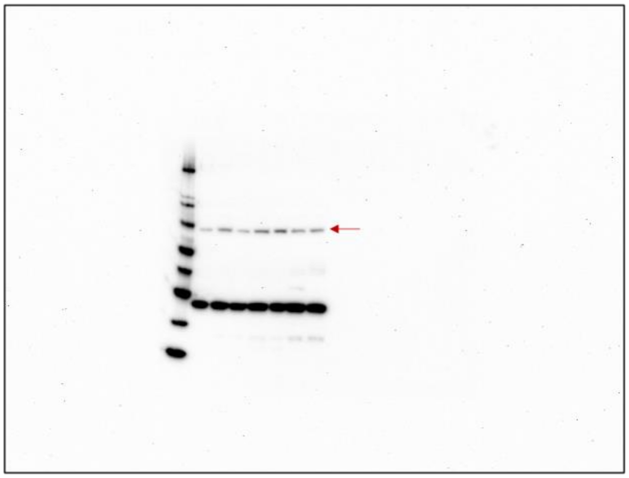

RIPK3, 60 kDA

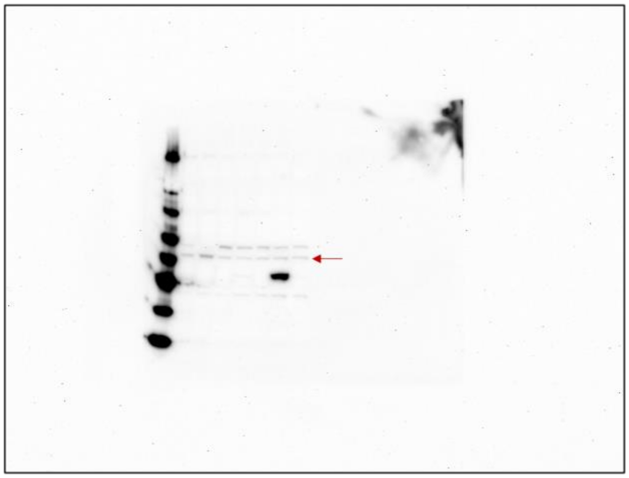

GAPDH, 37 kDa

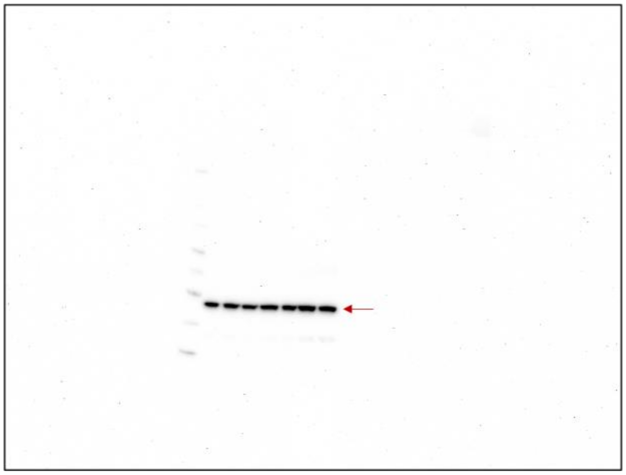

Membranes from Fig. 5:

OTC, 40 kDa

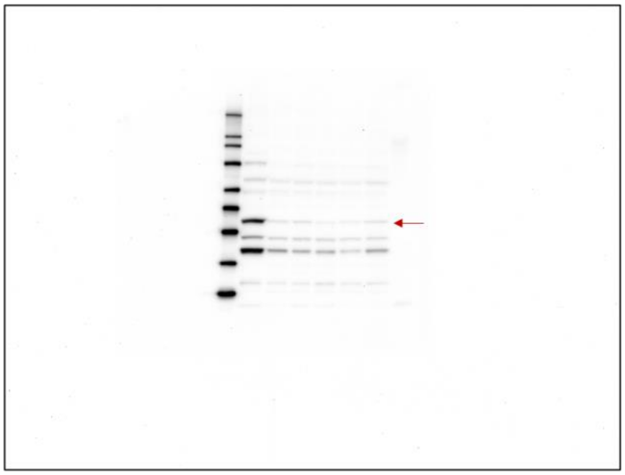

GAPDH, 37 kDa

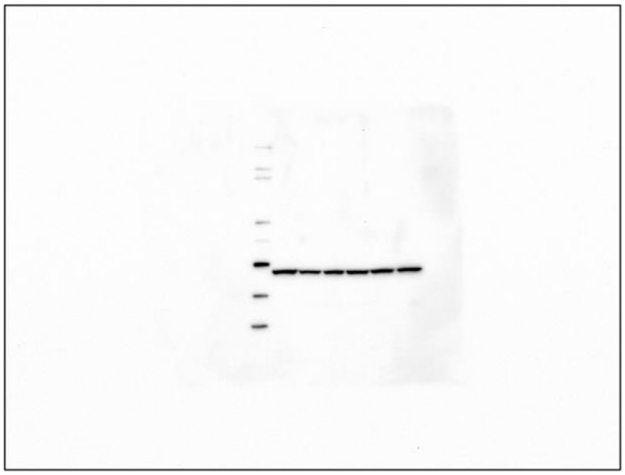

CPS1, 165 kDa

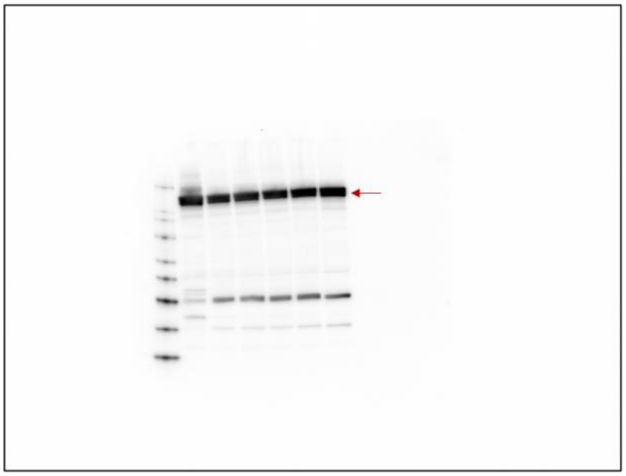

GAPDH, 37 kDa

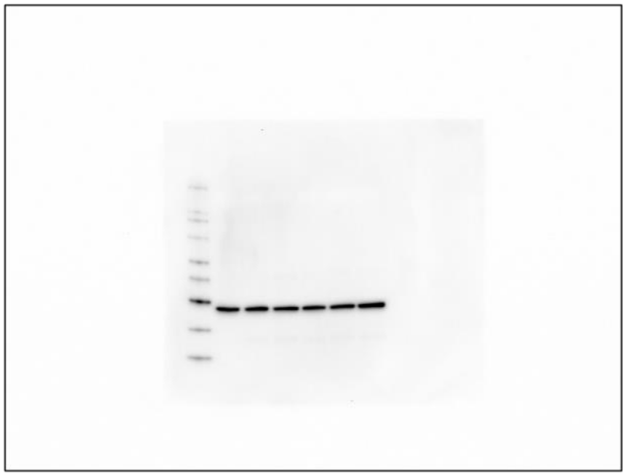

REFERENCES AND NOTES

1. D. Haussinger, Regulation of hepatic ammonia metabolism: The intercellular glutamine cycle. *Adv. Enzyme Regul.* **25**, 159–180 (1986).

2. D. Rudman, J. T. DiFulco, J. T. Galambos, R. B. D. Smith, A. A. Salam, W. D. Warren, Maximal rates of excretion and synthesis of urea in normal and cirrhotic subjects. *J. Clin. Invest.* **52**, 2241–2249 (1973).

3. S. Kaiser, W. Gerok, D. Haussinger, Ammonia and glutamine metabolism in human liver slices: New aspects on the pathogenesis of hyperammonaemia in chronic liver disease. *Eur. J. Clin. Invest.* **18**, 535–542 (1988).

4. A. Fabbri, G. Marchesini, G. Bianchi, E. Bugianesi, L. Bortoluzzi, M. Zoli, E. Pisi, Unresponsiveness of hepatic nitrogen metabolism to glucagon infusion in patients with cirrhosis: Dependence on liver cell failure. *Hepatology* **18**, 28–35 (1993).

5. R. E. Shangraw, F. Jahoor, Effect of liver disease and transplantation on urea synthesis in humans: Relationship to to acid-base status. *Am. J. Physiol.* **276**, G1145-52 (1999).

6. D. L. Shawcross, S. W. M. Olde Damink, R. F. Butterworth, R. Jalan, Ammonia and hepatic encephalopathy: The more things change, the more they remain the same. *Met. Brain Dis.* **20**, 169–179 (2005).

7. H. Vilstrup, P. Amodio, J. Bajaj, J. Cordoba, P. Ferenci, K. D. Mullen, K. Weissenborn, P. Wong, Hepatic encephalopathy in chronic liver disease: 2014 Practice guideline by the American Association for the Study of Liver Diseases and the European Association for the Study of the Liver. *Hepatology* **60**, 715–735 (2014).

8. C. R. Bosoi, C. F. Rose, Identifying the direct effects of ammonia on the brain. *Metab. Brain Dis.* **24**, 95–102 (2009).

9. D. L. Shawcross, G. A. K. Wright, V. Stadlbauer, S. J. Hodges, N. A. Davies, C. Wheeler-Jones, A. A. Pitsillides, R. Jalan, Ammonia impairs neutrophil phagocytic function in liver disease. *Hepatology* **48**, 1202–1212 (2008).

10. D. L. Shawcross, S. S. Shabbir, N. J. Taylor, R. D. Hughes, Ammonia and the neutrophil in the pathogenesis of hepatic encephalopathy in cirrhosis. *Hepatology* **51**, 1062–1069 (2010).

11. C. Luo, G. Shen, N. Liu, F. Gong, X. Wei, S. Yao, D. Liu, X. Teng, N. Ye, N. Zhang, X. Zhou, J. Li, L. Yang, X. Zhao, L. Yang, R. Xiang, Y. Wei, Ammonia drives dendritic cells into dysfunction. *J. Immunol.* **193**, 1080–1089 (2014).

12. G. Dam, P. Ott, N. Aagaard, H. Vilstrup, Branched-chain amino acids and muscle ammonia detoxification in cirrhosis. *Metab. Brain Dis.* **28**, 217–220 (2013).

13. S. Dasarathy, M. Hatzoglou, Hyperammonemia and proteostasis in cirrhosis. *Curr. Opin. Clin. Nutr. Metab. Care* **21**, 30–36 (2018).

14. J. Qiu, S. Thapaliya, A. Runkana, Y. Yang, C. Tsien, M. L. Mohan, A. Narayanan, B. Eghtesad, P. E. Mozdziak, C. McDonald, G. R. Stark, S. Welle, S. V. Naga Prasad, S. Dasarathy, Hyperammonemia in cirrhosis induces transcriptional regulation of myostatin by an NF-κB-mediated mechanism. *Proc. Natl. Acad. Sci. U.S.A.* **110**, 18162–18167 (2013).

15. G. Davuluri, A. Allawy, S. Thapaliya, J. H. Rennison, D. Singh, A. Kumar, Y. Sandlers, D. R. van Wagoner, C. A. Flask, C. Hoppel, T. Kasumov, S. Dasarathy, Hyperammonaemia-induced skeletal muscle mitochondrial dysfunction results in cataplerosis and oxidative stress. *J. Physiol.* **594**, 7341–7360 (2016).

16. A. Kumar, G. Davuluri, R. N. E. Silva, M. P. K. J. Engelen, G. A. M. ten Have, R. Prayson, N. E. P. Deutz, S. Dasarathy, Ammonia lowering reverses sarcopenia of cirrhosis by restoring skeletal muscle proteostasis. *Hepatology* **65**, 2045–2058 (2017).

17. F. De Chiara, K. L. Thomsen, A. Habtesion, H. Jones, N. Davies, J. Gracia-Sancho, N. Manicardi, A. Hall, F. Andreola, H. L. Paish, L. H. Reed, A. A. Watson, J. Leslie, F. Oakley, K. Rombouts, R. P. Mookerjee, J. Mann, R. Jalan, Ammonia scavenging prevents progression of fibrosis in experimental non-alcoholic fatty liver disease. *Hepatology* **71**, 874–892 (2020).

18. G. Ranucci, M. Rigoldi, G. Cotugno, S. M. Bernabei, A. Liguori, S. Gasperini, B. M. Goffredo, D. Martinelli, L. Monti, P. Francalanci, M. Candusso, R. Parini, C. Dionisi-Vici, Chronic liver involvement in urea cycle disorders. *J. Inherit. Met. Dis.* **42**, 1118–1127 (2019).

19. T. H. Tranah, M. P. Ballester, J. A. Carbonell-Asins, J. Ampuero, G. Alexandrino, A. Caracostea, Y. Sánchez-Torrijos, K. L. Thomsen, A. J. C. Kerbert, M. Capilla-Lozano, M. Romero-Gómez, D. Escudero-Carcía, C. Montoliu, R. Jalan, D. L. Shawcross, Plasma ammonia levels predict hospitalization with liver-related complications and mortality in clinically stable outpatients with cirrhosis. *J. Hepatol.* **77**, 1554–1563 (2022).

20. V. R. Patwardhan, Z. G. Jiang, Y. Risech-Neiman, G. Piatkowski, N. H. Afdhal, K. Mukamal, M. P. Curry, E. B. Tapper, Serum ammonia is associated with transplant-free survival in hospitalized patients with cirrhosis. *J. Clin. Gastroenterol.* **50**, 345–350 (2016).

21. Shalimar, M. F. Sheikh, R. P. Mookerjee, B. Agarwal, S. K. Acharya, R. Jalan, Prognostic role of ammonia in patients with cirrhosis. *Hepatology* **70**, 982–994 (2019).

22. C. Hu, K. Huang, L. Zhao, F. Zhang, Z. Wu, L. Li, Serum ammonia is a strong prognostic factor for patients with acute-on-chronic liver failure. *Sci. Rep.* **10**, 16970 (2020).

23. S. He, Y. Liang, F. Shao, X. Wang, Toll-like receptors activate programmed necrosis in macrophages through a receptor-interacting kinase-3-mediated pathway. *Proc. Natl. Acad. Sci. U.S.A.* **108**, 20054–20059 (2011).

24. A. R. Jayakumar, X. Y. Tong, K. M. Curtis, R. Ruiz-Cordero, M. T. Abreu, M. D. Norenberg, Increased toll-like receptor 4 in cerebral endothelial cells contributes to the astrocyte swelling and brain edema in acute hepatic encephalopathy. *J. Neurochem.* **128**, 890–903 (2014).

25. M. Jover-Cobos, L. Noiret, K. Lee, V. Sharma, A. Habtesion, M. Romero-Gomez, N. Davies, R. Jalan, Ornithine phenylacetate targets alterations in the expression and activity of glutamine synthase and glutaminase to reduce ammonia levels in bile duct ligated rates. *J. Hepatol.* **60**, 545–553 (2014).

26. C. Engelmann, M. Sheikh, S. Sharma, T. Kondo, H. Loeffler-Wirth, Y. B. Zheng, S. Novelli, A. Hall, A. J. C. Kerbert, J. Macnaughtan, R. Mookerjee, A. Habtesion, N. Davies, T. Ali, S. Gupta, F. Andreola, R. Jalan, Toll-like receptor 4 is a therapeutic target for prevention and treatment of liver failure. *J. Hepatol.* **73**, 102–112 (2020).

27. S. Matsumoto, J. Häberle, J. Kido, H. Mitsubuchi, F. Endo, K. Nakamura, Urea cycle disorders—Update. *J. Hum. Genet.* **64**, 833–847 (2019).

28. R. Jalan, F. De Chiara, V. Balasubramaniyan, F. Andreola, V. Khetan, M. Malago, M. Pinzani, R. P. Mookerjee, K. Rombouts, Ammonia produces pathological changes in human hepatic stellate cells and is a target for therapy of portal hypertension. *J. Hepatol.* **64**, 823–833 (2016).

29. S. C. S. Nagamani, S. Ali, R. Izem, D. Schady, P. Masand, B. L. Schneider, D. H. Leung, L. C. Burrage, Biomarkers of liver disease in urea cycle disorders. *Mol. Genet. Metab.* **133**, 148–156 (2021).

30. Y. K. Dhuriya, D. Sharma, Necroptosis: A regulated inflammatory mode of cell death. *J. Neuroinflammation* **15**, 199 (2018).

31. X. Li, G. Dong, H. Diao, A narrative review of necroptosis in liver disease: A double-edged sword. *Ann. Transl. Med.* **9**, 422 (2021).

32. T. Kondo, S. Macdonald, C. Engelmann, A. Habtesion, J. Macnaughtan, G. Mehta, R. P. Mookerjee, N. Davies, M. Pavesi, R. Moreau, P. Angeli, V. Arroyo, F. Andreola, R. Jalan, The role of RIPK1 mediated cell death in acute on chronic liver failure. *Cell Death Dis.* **13**, 5 (2022).

33. E. Seki, R. F. Schwabe, Hepatic inflammation and fibrosis: Functional links and key pathways. *Hepatology* **61**, 1066–1079 (2015).

34. E. Seki, S. de Minicis, C. H. Osterreicher, J. Kluwe, Y. Osawa, D. A. Brenner, R. F. Schwabe, TLR4 enhances TGF-β signaling and hepatic fibrosis. *Nat. Med.* **13**, 1324–1332 (2007).

35. M. E. Choi, D. R. Price, A. M. Choi, Necroptosis, a crucial pathogenic mediator of human disease. *JCI Insight* **4**, e128834 (2019).

36. J. Yaplito-Lee, C. Chow, A. Boneh, Histopathological findings in livers of patients with urea cycle disorders. *Mol. Genet. Metab.* **108**, 161–165 (2013).

37. B. Görg, A. Karababa, A. Shafigullina, H. J. Bidmon, D. Häussinger, Ammonia-induced senescence in cultured rat astrocytes and in human cerebral cortes in hepatic encephalopathy. *Glia* **63**, 37–50 (2015).

38. V. Felipo, R. F. Butterworth, Mitochondrial dysfunction in acute hyperammonemia. *Neurochem. Int.* **40**, 487–491 (2002).

39. P. R. Angelova, A. J. Kerbert, A. Habtesion, A. Hall, A. Y. Abramov, R. Jalan, Hyperammonaemia induces mitochondrial dysfunction and neuronal cell death. *JHEP Rep.* **4**, 100510 (2022).

40. M. Mercado-Gómez, N. Goikoetxea-Usandizaga, A. J. C. Kerbert, L. U. Gracianteparaluceta, M. Serrano-Macía, S. Lachiondo-Ortega, R. Rodriguez-Agudo, C. Gil-Pitarch, J. Simón, I. González-Recio, M. F. Fondevila, P. Santamarina-Ojeda, M. F. Fraga, R. Nogueiras, J. de Las Heras, R. Jalan, M. L. Martinéz-Chantar, T. C. Delgado, The lipopolysaccharide-TLR4 axis regulates glutaminase 1 expression promoting liver ammonia build-up as steatotic liver disease progresses to steatohepatitis. *Metabolism* **158**, 155952 (2024).

41. R. Jalan, S. W. M. Olde Damink, H. F. Lui, M. Glabus, N. E. P. Deuts, P. C. Hayes, K. Ebmeier, Oral amino acid load mimicking hemoglobine results in reduced regional cerebral perfusion and deterioration in memory tests in patients with cirrhosis of the liver. *Metab. Brain Dis.* **18**, 37–49 (2003).

42. G. Allegri, S. Deplazes, N. Rimann, B. Causton, T. Scherer, J. W. Leff, C. Diez-Fernandez, A. Klimovskaia, R. Fingerhut, J. Krijt, V. Kozich, J. Nuoffer, H. M. Grisch-Chan, B. Thöny, J. Häberle, Comprehensive characterization of ureagenesis in the spf^{ash} mouse, a model of human ornithine transcarbamylase deficiency, reveals age-dependency of ammonia detoxification. *J. Inherit. Metab. Dis.* **42**, 1064–1076 (2019).

43. H. M. H. van Eijk, D. R. Rooyakkers, N. E. P. Deutz, Rapid routine determination of amino acids in plasma by high-performance liquid chromatography with a 2–3 μm Spherisorb ODS II column. *J. Chromatography* **620**, 143–148 (1993).

44. A. Guillot, M. Kohlhepp, A. Bruneau, F. Heymann, F. Tacke, Deciphering the immune microenvironment on a single archival formalin-fixed paraffin-embedded tissue section by an immediately implementable multiplex fluorescence immunostaining protocol. *Cancers (Basel)* **12**, 2449 (2020).

45. P. B. Saint Hillaire, K. Rousseau, A. Seyer, S. Dechaumet, A. Damont, C. Junot, F. Fenaille, Comparative envaluation of data dependent and data independent acquisition workflows implemented on an orbitrap fusion for untargeted metabolomics. *Metabolites* **10**, 158 (2020).

46. A. Imbert, M. Rompais, M. Selloum, F. Castelli, E. Mouton-Barbosa, M. Brandolini-Bunlon, E. Chu-Van, C. Joly, A. Hirschler, P. Roger, T. Burger, S. Leblanc, T. Sorg, S. Ouzia, Y. Vandenbrouck, C. Médigue, C. Junot, M. Ferro, E. Pujos-Guillot, A. Gonzalez de Peredo, F. Fenaille, C. Carapito, Y. Herault, E. A. Thévenot, ProMetIS, deep phenotyping of mouse models by combined proteomics and metabolomics analysis. *Sci. Data* **8**, 311 (2021).

47. S. Boudah, M. Olivier, S. Aros-Calt, L. Oliveira, F. Fenaille, J. Tabet, C. Junot, Annotation of the human serum metabolome by coupling three liquid chromatography methods to high-resolution mass spectrometry. *J. Chromatogr. B Analyt. Technol. Biomed. Life Sci.* **966**, 34–47 (2014).

48. F. Giacomoni, G. Le Corguillé, M. Monsoor, M. Landi, P. Pericard, M. Pétéra, C. Duperier, M. Tremblay-Franco, J. Martin, D. Jacob, S. Goulitquer, E. A. Thévenot, C. Caron, Workflow4Metabolomics: A collaborative research infrastructure for computational metabolomics. *Bioinformatics* **31**, 1493–1495 (2015).

49. H. Tsugawa, T. Cajka, T. Kind, Y. Ma, B. Higgins, K. Ikeda, M. Kanazawa, J. VanderGheynst, O. Fiehn, A. Masanori, MS-DIAL: Data-independent MS/MS deconvolution for comprehensive metabolome analysis. *Nat. Methods* **12**, 523–526 (2015).

50. L. W. Sumner, A. Amberg, D. Barret, M. H. Beale, R. Beger, C. A. Daykin, T. W. M. Fan, O. Fiehn, R. Goodacre, J. L. Griffin, T. Hankemeier, N. Hardy, J. Harnly, R. Higashi, J. Kopka, A. N. Lane, J. C. Lindon, P. Marriott, A. W. Nicholls, M. D. Reilly, J. J. Thaden, M. R. Viant, Proposed minimum reporting standards for chemical analysis Chemical Analysis Working Group (CAWG) Metabolomics Standards Initiative (MSI). *Metabolomics* **3**, 211–221 (2007).

51. M. Charni-Natan, I. Goldstein, Protocol for primary mouse hepatocyte isolation. *STAR Protoc.* **1**, 100086 (2020).

52. P. Kumar, M. Hassan, F. Tacke, C. Engelmann, Delineating the heterogeneity of senescence-induced-functional alterations in hepatocytes. *Cell. Mol. Life Sci.* **81**, 200 (2024).

53. N. Nikolac, J. Omazic, A. Simundic, The evidence based practice for optimal sample quality for ammonia measurement. *Clin. Biochem.* **47**, 991–995 (2014).

54. F. Da Foncesca-Wollheim, Preanalytical increase of ammonia in blood specimens from healthy subjects. *Clin. Chem.* **36**, 1483–1487 (1990).

55. Z. Pang, J. Chong, G. Zhou, D. A. de Lima Morais, L. Chang, M. Barrette, C. Gauthier, P. Jacques, S. Li, J. Xia, MetaboAnalyst 5.0: Narrowing the gap between raw spectra and functional insights. *Nucleic Acids Res.* **49**, W388–W396 (2021).

56. S. Chopra, H. M. Wallace, Induction of spermidine/spermine N^1-acetyltransferase in human cancer cells in response to increased production of reactive oxygen species. *Biochem. Pharmacol.* **55**, 1119–1123 (1998).

57. M. M. Corvi, C. L. Soltys, L. G. Berthiaume, Regulation of mitochondrial carbamoyl-phosphate synthetase 1 activity by active site fatty acylation. *J. Biol. Chem.* **276**, 45704–45712 (2001).

58. B. E. Corkey, D. E. Hale, M. C. Glennon, R. I. Kelley, P. M. Coates, L. Kilpatrick, C. A. Stanley, Relationship between unusual hepatic acyl coenzyme A profiles and the pathogenesis of Reye syndrome. *J. Clin. Invest.* **82**, 782–788 (1988).

59. M. Costell, J. E. O'Connor, M. P. Miguez, S. Grisolia, Effects of L-carnitine on urea synthesis following acute ammonia intoxication in mice. *Biochem. Biophys. Res. Commun.* **120**, 726–733 (1984).

60. M. R. McGill, F. Li, M. R. Sharpe, C. David Williams, S. C. Curry, X. Ma, H. Jaeschke, Circulating acylcarnitines as biomarkers of mitochondrial dysfunction after acetaminophen overdose in mice and humans. *Arch. Toxicol.* **88**, 391–401 (2014).

61. D. M. Muoio, Metabolic inflexibility: When mitochondrial indecision leads to metabolic gridlock. *Cell* **159**, 1253–1262 (2014).

62. W. G. Hunter, J. P. Kelly, R. W. McGarrahlll, M. G. Khouri, D. Crag, C. Haynes, O. Ilkayeva, R. D. Stevens, J. R. Bain, M. J. Muehlbauer, C. B. Newgard, G. M. Felker, A. F. Hernandez, E. J. Velazquez, W. E. Kraus, S. H. Shah, Metabolomic profiling identifies novel circulating biomarkers of mitochondrial dysfunction differentially elevated in heart failure with preserved versus reduced ejection fraction: Evidence for shared metabolic impairments in clinical heart failure. *J. Am. Heart Assoc.* **5**, e003190 (2016).

63. A. Atlante, S. Passarella, S. Giannattasio, E. Quagliariello, Fumarate permeation in rat liver mitochondria: Fumarate/malate and fumarate/phosphate translocators. *Biochem. Biophys. Res. Commun.* **132**, 8–18 (1985).

64. Z. Alexandrowicz, J. Swierczynski, Fumarate transport by rat liver mitochondria. *FEBS Lett.* **15**, 269–272 (1971).
